# Design and Synthesis of Polyheterocyclic Compounds Containing Pyrazolopyridopyrimidine Nucleus with Antimicrobial Activities

**DOI:** 10.1002/open.202400070

**Published:** 2024-04-29

**Authors:** Farag A. El‐Essawy, Mohammad Ahmad Ahmad Odah

**Affiliations:** ^1^ Preparatory Year Deanship. Basic Science Department Prince Sattam Bin Abdulaziz University, 151 Alkharj 11942, KSA Saudi Arabia

**Keywords:** Cyclocondesation, Pyrazolopyridopyrimidine, Polyheterocyclic, Thiourea, Antimicrobial

## Abstract

This study reports the design, synthesis, and antibacterial evaluation of a library of novel polyheterocyclic derivatives featuring a unique fused pyrimidopyridopyrazole moiety. A cyclocondensation reaction between an amino−pyrazolopyridopyrimidine precursor and malonates afforded a series of pyrimidopyridopyrazolopyrimidine derivatives. Further diversification was achieved through nucleophilic cyclocondensation, yielding a collection of complex polyheterocyclic systems encompassing various ring structures. All synthesized compounds were rigorously characterized using spectroscopic techniques and elemental analysis. The antibacterial activity of the newly synthesized compounds was assessed against a panel of Gram‐positive and Gram‐negative bacteria. Notably, several compounds exhibited promising antibacterial activity, highlighting their potential as leads for the development of novel antibiotics.

## Graphical abstract

This work introduces novel polyheterocyclic derivatives featuring a fused pyrimidopyridopyrazole core, synthesized through cyclocondensation reactions with amino‐pyrazolopyridopyrimidine precursors and various malonates. These compounds, characterized by spectroscopic techniques, exhibit significant antibacterial activity against both Gram‐positive and Gram‐negative bacteria, underscoring their potential as novel antibiotic leads.

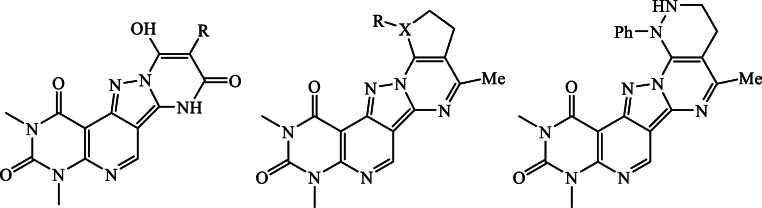

## Introduction

**Figure 1:**
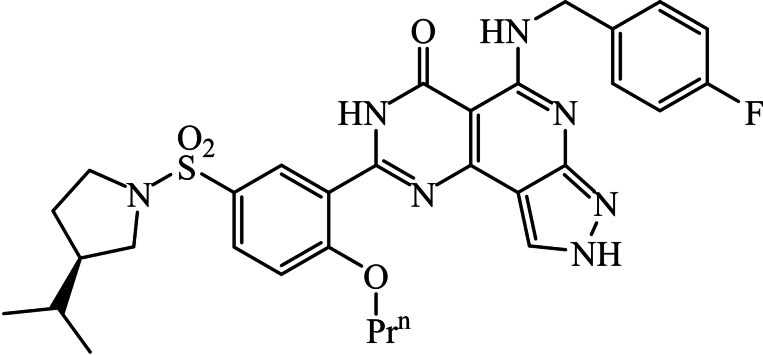
Pharmaceutical compound containing Pyrazolopyridopyrimidine ring system.

Heterocyclic compounds and their nitrogen derivatives are esteemed for their diverse and potent biological and pharmacological properties, encompassing antimicrobial, antifungal, antimalarial, anti‐inflammatory, analgesic, and potential anticancer activities.^[1]^ Furthermore, Pyridine, a vital *N*‐heteroaromatic structure, plays a central role in the design of therapeutic agents, displaying compelling biological effects. These pyridine‐based heterocyclic compounds also serve as crucial intermediates in synthesizing a wide range of bioactive molecules.^[2]^ They occur naturally in antibiotics, nucleic acid, hormones, vitamins, and other substances.^[3]^ As a result, studies on synthesis are being conducted of poly‐functionalized compounds, there has been a lot of interest in heterocyclic compounds that are crucial to the drug discovery process. Heterocycles with pyrazolo annulation, like pyrazolopyridopyrimidines, which have five nitrogen atoms in one molecule and three fused heterocyclic skeletons, may combine the characteristics of all three fused heterocycles and show a variety of uses in the textile and agricultural industries,^[4]^ and the medical sector, such as anti‐proliferative agents.^[5]^ These compounds exhibit a variety of biological traits, including virucidal, anticancer, fungicidal, bactericidal, and vasodilator activities.[^6^, ^7^] Additionally, pyrazolopyridopyrimidine derivatives demonstrated strong antitumor effects and high antibacterial activities.^[8]^ Figure 1 presents some significant pyrazolopyridopyrimidine scaffolds from a biological perspective.[^9^, ^10^, ^11^]

**Figure 2:**
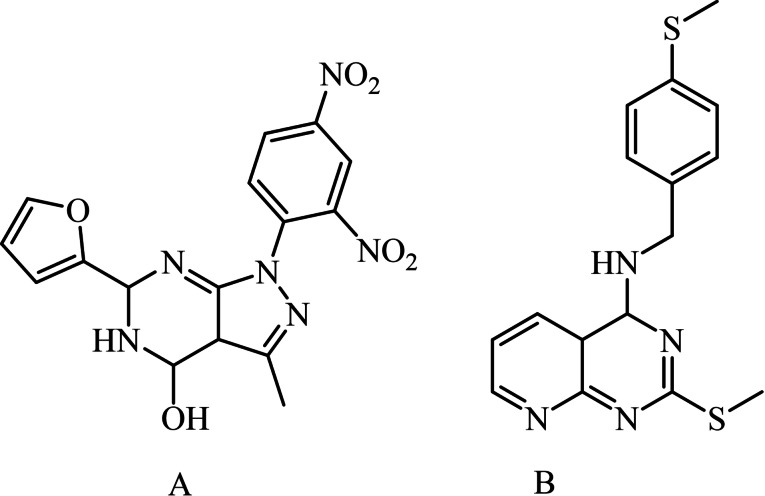
Cytotoxic compounds previously reported: pyrazole‐fused pyrimidine A and pyridine‐fused pyrimidine B.

The results from the literature previously reported indicate that designing new chemical entities with increased activity may be facilitated by combining two or more bioactive heterocyclic pharmacophores into a single molecule. As a result, pyrazole‐fused pyrimidines are a versatile and promising class of heterocyclic scaffolds that have always piqued the interest of chemists due to their exceptional potential, such as antitumor properties,^[12]^ anti‐inflammatory,^[13]^ anti‐cancer and anti‐5‐lipoxygenase,^[14]^ anti‐bacterial,^[15]^ anti‐tubercular^[16]^ and particular cytotoxic properties.[^17^, ^18^, ^19^, ^20^] Figure 2.

Pyrido−pyrimidines have long piqued the interest of biological and polymeric researchers because of their numerous biological therapeutic properties, including antiepileptic,^[21]^ antimutagenic,^[22]^ anti‐proliferative,^[23]^ fungicidal,^[24]^ adenosine kinase suppression,^[25]^ and anticancer properties,[^26^, ^27^] as shown in Figure 2.

Motivated by the aforementioned findings, and in resumption of our prior projects[^28^, ^29^, ^30^] on the synthesis of new fused‐pyrimidine scaffolds, we wanted to create new pyrazolo[4,3‐e]pyrido[1,2‐a]pyrimidines compounds derivatives of fused with six and five membered heterocyclic rings with antibacterial activity testing.

## Results and Discussion

**Scheme 1:**
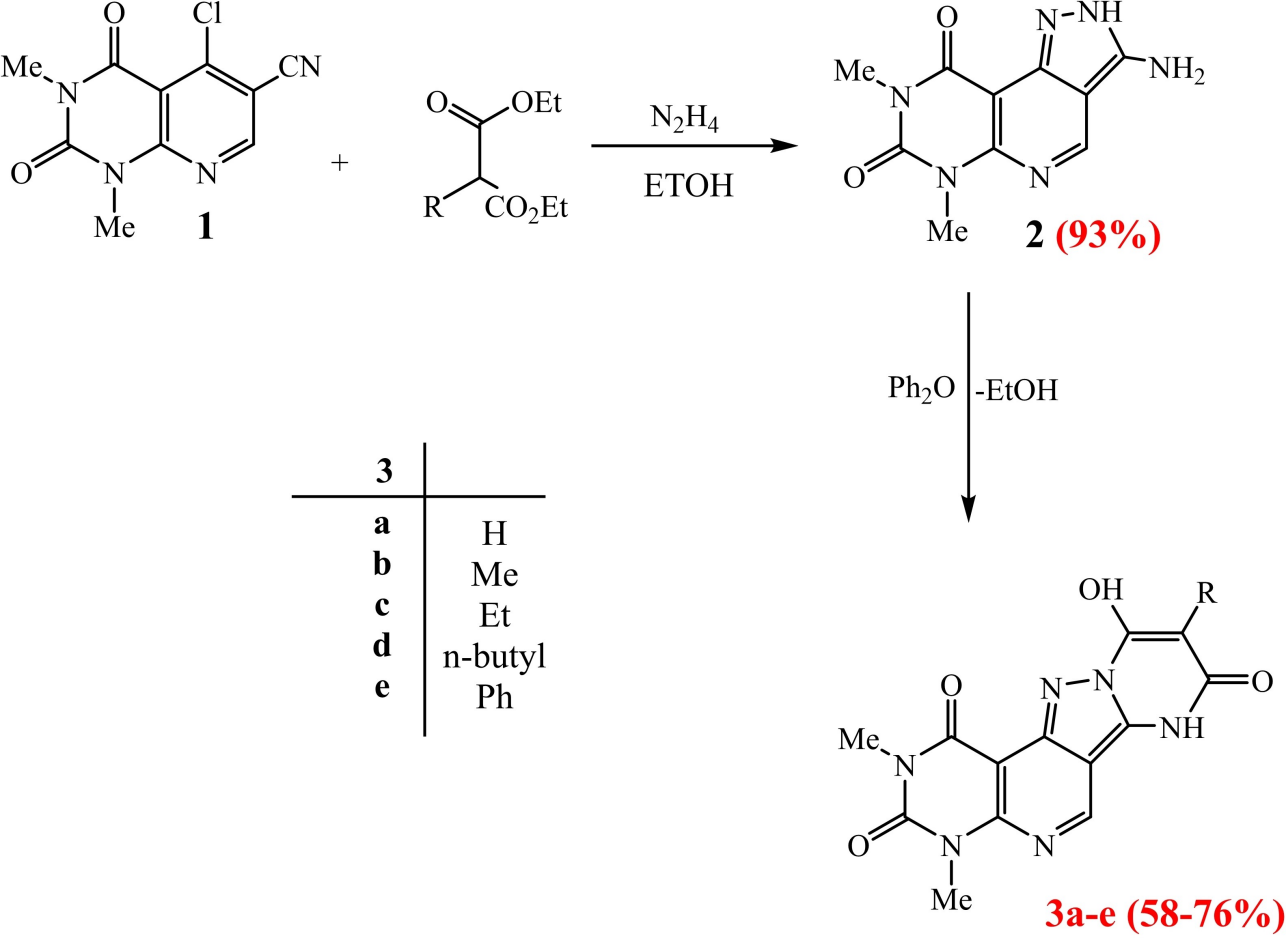
Synthesis of the Tetraheterocyclic systems.

We selected 5‐chloro‐1,3‐dimethyl‐2,4‐dioxo‐1,2,3,4‐tetrahydropyrido[2,3‐d]pyrimidine‐6‐carbonitrile (**1**) which was prepared according to the reported method.^[31]^ The choro derivative **1** was used to synthesis of the starting material after its treatment with hydrazine hydrate (80 %) to afford 3‐amino‐6,8‐dimethyl‐2H‐pyrazolo[3′,4′:4,5]pyrido[2,3‐d]pyrimidine‐7,9(6H,8H)‐dione (**2**) which was used to synthesis the tetraheterocyclic systems Scheme (1). To construct a new pyrimidine ring fused with pyrazolo[3′,4′:4,5]pyrido[2,3‐d]pyrimidine derivative **2**, the amino derivative 2 was subjected to the cyclocondensation reactions with malonate derivatives, in diphenyl ether at 250 °C, affording the corresponding pyrimido[5′′′,4′′′:5′′,6′′]pyrido[4′′,3′′:3′,4′]pyrazolo[1′,5′:1,2]pyrimidine derivatives **3 a**–**e**, according to the mechanism shown in scheme 1. The structure of the synthesized derivatives **3 a**–**e** were confirmed using mass spectrometry and NMR. The structure of the starting derivative **2** was obviously confirmed by ^1^H‐NMR spectrum, which showed the presence of the single signals at δ 7.69 ppm, related to the NH_2_ and NH of pyrazole ring respectively, as well as in the IR spectrum showed the disappearance of the characteristic peak related to CN group (2150 cm^−1^), but the appearance of a single peaks at 3265 cm^−1^ related to NH, NH_2_ groups. The structure of derivatives **3 a**–**e** were clearly confirmed by the spectral data. ^1^H‐NMR spectra of derivatives **3 a**–**e** indicated the appearance of new single signals at δ 9.30 ppm and 10.12–13.12 ppm for NH, and OH groups respectively of new pyrimidine ring formation, also, appearance of the new signals corresponding to proton (Ar), methyl, ethyl, n‐butyl and/or phenyl groups at carbone‐9. Besides, the MS spectrum revealed the molecular ion peaks at the exact molecular weight of the derivatives **3 a**–**e**.

**Scheme 2:**
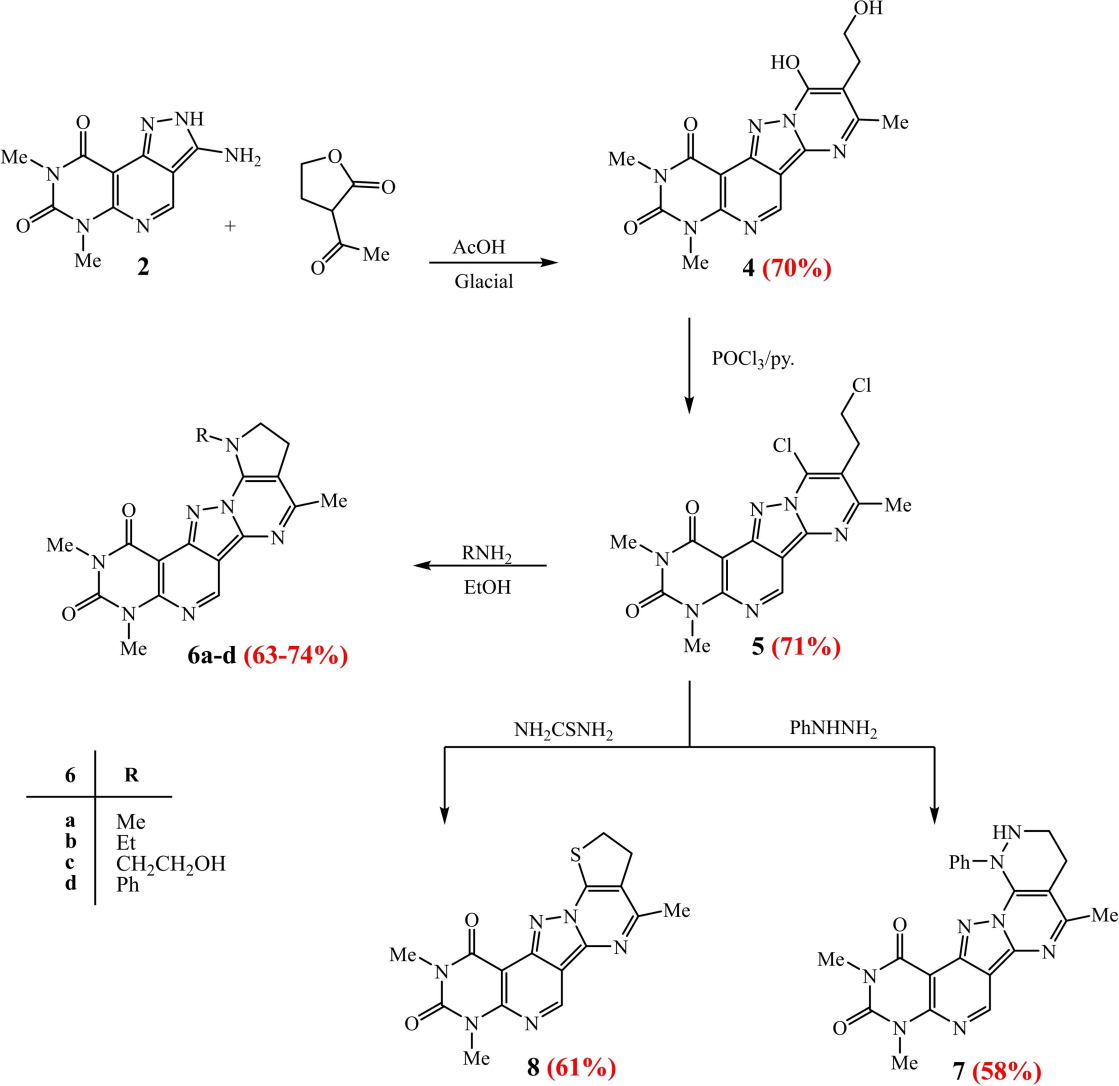
Synthesis of fused Pentaheterocyclic systems.

On treatment of aminopyrazole derivative **2** with 3‐acetyldihydrofuran‐2(3H)‐one, in glacial acetic acid, undergo the nucleophilic addition followed by cyclocondensation reactions, according to the reaction mechanism shown in scheme 2, affording the tetracyclic derivatives **4**, in good yield 70 %. The dihydroxy derivative **4** was chlorinated after its treatment with POCl_3_/py. to give the dichloro derivative **5**. Their IR spectra showed a three peaks characteristics for carbonyl groups absorption at 1630, 1644, 1688 cm^−1^ for derivative **4**, but only two peaks at 1658, 1690 cm^−1^ corresponding to CO groups for derivative **5**, indicating the chlorination reaction occurred at carbon (C‐8) in derivative **5**. Their ^1^H‐NMR spectra agree with the structure that showed a characteristics triplet‐triplet for CH_2_−CH_2_−OH at δ 277, 3.52 ppm for **4** but triplet‐triplet CH_2_−CH_2_−Cl at δ 3.44, 3.96 ppm for **5**. Where 9‐(2‐Chloroethyl)‐8‐chloro‐2,4‐dimethyl‐pyrimido[5′′′,4′′′:5′′,6′′]pyrido[4′′,3′′:3′,4′]pyrazolo[1′,5′:1,2]pyrimidine‐1,3,(2H,4H)‐dione (**5**) serves as a key intermediate for the synthesis of polyheterocyclic system (five condensed rings) after its treatment with the appropriate primary amine in absolute ethanol, under basic condition, to give pyrimido[5′′′,4′′′:5′′,6′′]pyrido[4′′,3′′:3′,4′]pyrazolo[1′,5′:1,2]pyrimido[4,5‐c] pyrrolidine derivatives **6 a**–**d** scheme 2.

The mechanism of the nucleophile cyclocondensation reactions (as shown in scheme 2) proceed through the nucleophilic attack of the primary amine on the electrophilic carbons, bearing chloride, results in dehyrochlorination (−2HCl) with formation of trihydropyrimido[5′′′,4′′′:5′′,6′′]pyrido[4′′,3′′:3′,4′]pyrazolo[1′,5′:1,2]pyrimido[4,5‐c] pyrrolidine‐1,3(2H,4H)‐dione (**6 a**–**d**). The structures of these compounds **6 a**–**d** were confirmed by ^1^H NMR, IR, MS and elemental analysis. Their ^1^H NMR revealed a new signals related the *N*‐alkyl (Me, Et, CH_2_CH_2_OH) for derivatives **6 a**–**c** but *N*‐Ph for derivative **6 d** respectively. In addition, the dichloro derivative **5** undergoes nucleophile cyclocondensation reactions on its treatment with phenylhydrazine and thiophene, in in abs. ethanol, to afford the pyridazino **7** and thiopheno **8** derivatives respectively. The structure of the derivatives **7**, **8** have been verified by NMR and MS spectroscopy. ^1^H‐NMR spectrum of derivative **7** present at δ 7.15–7.36 ppm corresponding to the *N*‐phenyl group of new pyridazine ring formation, besides a single signal at δ 10.23 ppm related to NH group. But on the other hand, the MS spectrum of derivative **8** revealed the molecular ion peak at 354, corresponding the molecular weight (Scheme 2).

### Biological Activity Assessment

In our investigation of novel antibacterial compounds (3a–e, 4, 5, 6a–d, 7, and 8), we employed a broth microdilution method to assess their efficacy against four prevalent bacterial strains. This method offers a quantitative measure of a compound′s antibacterial activity by determining the minimum inhibitory concentration (MIC) and sub‐MIC values.^[32]^ The MIC represents the lowest concentration of the compound that completely inhibits visible bacterial growth after a defined incubation period.^[33]^ Sub‐MICs, on the other hand, represent concentrations below the MIC that still exert a measurable effect on bacterial growth.[^34^, ^35^]

We employed the following protocol to determine the MIC and sub‐MIC values:

1. Serial dilutions of each compound were prepared in a cation‐adjusted Mueller‐Hinton broth (CAMHB) to achieve a broad range of concentrations.
2. Standardized inocula of each bacterial strain were prepared and added to wells containing the diluted compounds.
3. The plates were incubated at 37°C for 24 hours (bacteria) to allow for bacterial growth.
4. The MIC was determined as the lowest concentration of the compound that resulted in no visible bacterial turbidity compared to the growth control (bacteria with no test compound).
5. Sub‐MICs were determined by measuring the extent of bacterial growth inhibition at concentrations below the MIC.

Ampicillin, a broad‐spectrum antibiotic, was incorporated as a positive control in each assay. This ensures the validity of the assay by verifying that the bacteria are viable and susceptible to established antibiotics.^[34]^ By comparing the activity of our test compounds to Ampicillin, we gain valuable context for interpreting their potency.[^35^, ^36^]

### Antimicrobial Activity Evaluation

This study evaluated the antibacterial efficacy of thirteen synthesized compounds (3a–e, 4, 5, 6a–d, 7, and 8) against four bacterial strains: *Escherichia coli* (Gram‐negative), *Pseudomonas aeruginosa* (Gram‐negative), *Staphylococcus aureus* (Gram‐positive), and *Staphylococcus pyogenes* (Gram‐positive). The assessment employed sub‐Minimum Inhibition Concentrations (sub‐MICs), which are presented in Table 1.

**Table 1:** The antimicrobial potency of the tested compounds.

Comp. no.		Sub‐Minimum inhibition concentrations (sub‐MICs)		
		For bacteria (sub‐MICs) in μg ml^−1^		
	Gram‐negative		Gram‐positive	
	*Escherichia* *coli*	*Pseudomonas* *aeruginosa*	*Staphylococcus* *aureus*	*Staphylococcus* *pyogenes*
3a	125	250	250	250
3b	250	125	<500	125
**3 c**	**50**	**50**	**6.25**	**50**
**3 d**	**50**	**50**	**6.25**	**50**
**3 e**	125	125	125	50
**4**	**25**	**6.25**	**50**	**25**
5	<500	250	500	250
**6 a**	**25**	**6.25**	**50**	**25**
**6 b**	**25**	**6.25**	**50**	**25**
**6 c**	**25**	**6.25**	**50**	**25**
**6 d**	**25**	**6.25**	**50**	**25**
7	250	125	<500	125
**8**	**50**	**50**	**6.25**	**50**
Ampicillin	**50**	**50**	**50**	**50**

E.c *Escherchia coli* MTCC 443; P.a *Pseudomonas aeruginosa* MTCC 1688; S. a *Staphylococcus aureus* MTCC 96; S. p. *Staphlococcus pygenes* MTCC 442. **Most potent compounds are indicated by bold values**.

Based on the sub‐MICs in Table 1, compounds 6a–d, 4, and 8 emerged as the most potent candidates, exhibiting consistently lower values across various bacteria. This signifies their superior broad‐spectrum antibacterial activity.

Detailed Observations by Bacteria:

*Escherichia coli*: Compounds 4 and 6a–d demonstrated the strongest inhibition, with compound 4 having the lowest sub‐MIC (25 μg ml^−1^).

*Pseudomonas aeruginosa*: Compounds 8 and 4 displayed notable inhibition, with sub‐MICs comparable to the reference antibiotic Ampicillin (Table 1). Conversely, compound 5 exhibited lower potency against this bacterium.

*Staphylococcus aureus*: Compounds 3c, 3d, 4, 6a–d, and 8 displayed effective inhibition, with compound 4 again demonstrating the strongest activity (sub‐MIC of 6.25 μg ml^−1^).

**Figure 3:**
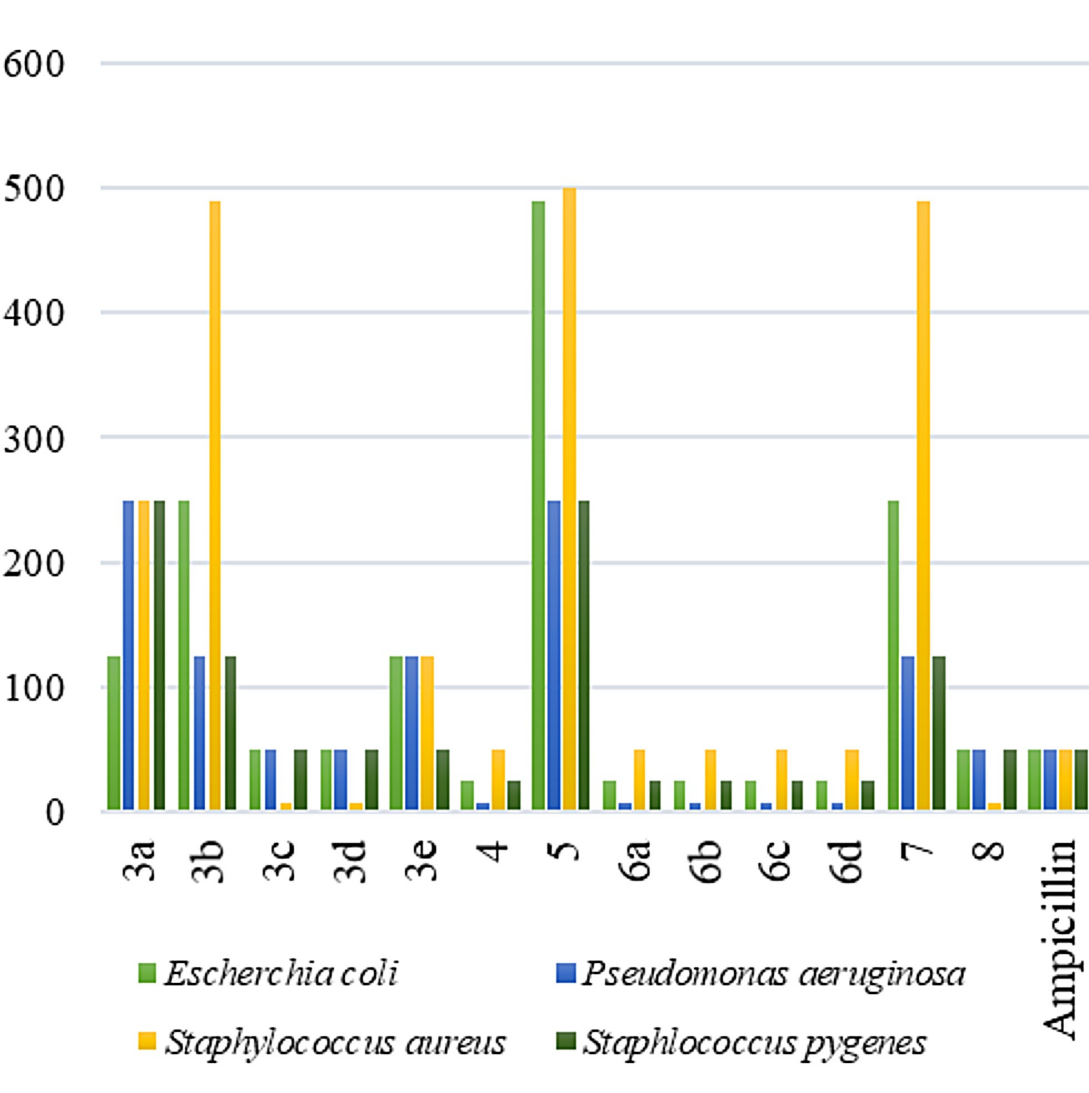
antimicrobial potency of tested compounds.

*Staphylococcus pyogenes*: Compounds 6a–d and 8 exhibited good activity, while compound 5 showed the highest inhibition (sub‐MIC of 6.25 μg ml^−1^) as shown in Figure 3.

### Structure‐Activity Relationship Study

The study reveals potent antibacterial activity within the tested compounds, with particular strength exhibited by **3 c**, 3d, 4, 6a–d, and 8. This efficacy can be traced back to the intricate molecular structures and functional groups present. Several key features likely contribute to their ability to combat bacteria:

These aromatic moieties may interact with essential bacterial enzymes or receptors, disrupting crucial cellular processes. This complexity enhances the surface area available for binding to bacterial targets, potentially increasing potency. Substituents like phenyl, ethyl, and hydroxyethyl on the aromatic rings influence the molecule‘s interaction with bacterial components by modulating its hydrophobicity and hydrophilicity. In compounds 6a–d, the presence of a pyrrolidine ring and the unique pyrimido−pyrido−pyrazolo core further elevate the molecular complexity, offering additional sites for interaction with bacterial targets. Additionally, the diverse substituents (methyl, ethyl, hydroxyethyl, and phenyl) contribute to a broader spectrum of antibacterial activity by tailoring the chemical structure to specific bacterial strains. Compound contain pyridazine ring adds another layer of structural intricacy. The methyl and phenyl substituents on this ring likely influence the compound‘s lipophilicity, impacting its ability to penetrate bacterial membranes.

Ultimately, the observed antibacterial activity arises from a confluence of factors. Functional groups like imino and hydroxyl groups facilitate hydrogen bonding with bacterial components, while the overall molecular architecture and the presence of polyheteroaromatic systems provide ample opportunities for targeted interactions. This interplay between structure and function, combined with the diversity within the tested compounds, leads to varying levels of potency against different bacterial strains, paving the way for more targeted and effective antibacterial strategies.

## Experimental

All compounds were obtained commercially and were utilized without any further purifying. A Kofter block instrument was used to determine melting points (mp). TLC (silica gel 60F254, Merck, Darmstadt, Germany) monitored reactions. ^1^H and ^13^C NMR spectra were measured on the standard apparatus (300 MHz, 75.5 MHz) using DMSO‐*d_6_* or CDCl_3_ to dissolve samples. A GC MS‐QP 1000 EX Mass Spectrometer was used to obtain mass spectra (Shimadzu, Tokyo, Japan), Cairo University's Faculty of Science's Microanalytical Laboratory. Elemental analyses were carried out in the microanalysis laboratory at Cairo University using a Yanaca CHN Corder MT‐3 elemental analyzer. The IR spectra have been captured using KBr disks on a Perkin‐Elmer 1720 FT‐IR spectrometer. Antibacterial potency was assessed at Prince Sattam Bin Abdul‐Aziz University's College of Pharmacy, Department of Pharmaceutics.

### 3‐Amino‐6,8‐dimethyl‐2,6‐dihydro‐7H‐pyrazolo[3′,4′:4,5]pyrido[2,3‐d]pyrimidine‐7,9(8H)‐dione (2)

To a clear solution of chloro derivative **1** (2.5 g, 10 mmol) in 15 ml absolute ethanol, 80 % hydrazine hydrate (2.5 g 50 mmol) was added in one portion. The mixture was heated under reflux for 5 h. until complete the reaction (TLC). After cooling, the solid product was collected by filtration, deride and recrystallized from ethanol to get the amino pyrazole derivative **2** in good yield 2.3 g, 93 %, mp 153–155 °C .IR (KBr), ν, cm^−1^: 3320–3210 (NH, NH_2_), 1650, 1695 (2‐CO). ^1^H NMR (DMSO‐*d_6_*), δ, ppm: 3.15, 3.35 (2 s, 6H, 2 NCH_3_), 5.32 (bs, 2H, NH_2_); 8.14 (s, 1H, Harom), 10.07 (1H, bs, NH). ^13^C NMR (CDCl_3_), δc, ppm: 24.2, 27.6 (2 N CH_3_), 132.6, 141.3, 142.4, 148.8, 150.6, 151.2 (6 C arom), 154.5, 159.8 (2 C=O). Mass spectrum (EI, 70 eV), *m/z* (*I*_ref_, %): 246 (M^+^, 10), 216 (15), 201 (72), 43 (100). Found %: C, 48.19; H, 3.66; N, 34.03. C_10_H_10_N_6_O_2_. (246.09) Calculated %: C, 48.78; H, 4.09; N, 34.13.

### General Procedure for Preparation of Pyrimido[5′′′,4′′′:5′′,6′′]pyrido[4′′,3′′:3′,4′]pyrazolo[1′,5′:1,2]pyrimidine Derivatives (3 a–e)

To a solution of aminopyrazole **2** (2.46 g, 10 mmol) in diphenyl ether (10 ml), an appropriate malonate derivative RCH(COOEt)_2_ (10 mmol) was added. The mixture was refluxed in an oil bath for 20–30 min at temperature 220–223 °C using a short air condenser to remove the liberated ethanol. Then the reaction cooled and triturated with diethyl ether to obtain the precipitate which filtered, washed with diethyl ether, dried, and recrystallized from acetic acid, giving a yellow derivatives **3 a**–**e**.

### 10‐Hydroxy‐2,4‐dimethyl‐7,8‐dihydropyrimido[5′′′,4′′′:5′′,6′′]pyrido[4′′,3′′:3′,4′]pyrazolo[1′,5′:1,2]pyrimidine‐1,3,8(2H,4H, 7H)‐trione (3 a)

Yield, 2.3 g 73 %; mp 298–301 °C; IR (KBr), ν, cm^−1^: 3200–3075 (OH, NH), 1665 (C=O). ^1^H NMR (DMSO‐*d_6_*), δ, ppm (*J*, Hz): 2.86, 3.18 (6H, s, 2 NCH_3_), 7.77, 8.12 (2H, s, HAr), 9.88 (^1^H, bs, NH), 11.12 (^1^H, bs, OH). ^13^C NMR (DMSO‐*d_6_*), δc, ppm: 27.3, 29.5 (2 N CH_3_), 114.2, 132.4, 138.7, 140.6, 141.4, 149.7, 150.6, 153.8, (10 C Ar), 155.2, 158.5, 161.3 (3 C=O). Mass spectrum (EI, 70 eV), *m/z* (*I*_ref_, %): 314 (M^+^, 2), 280 (70), 266 (100). Found %: C, 49.49; H, 3.15; N, 26.63. C_13_H_10_N_6_O_4_ (314.26). Calculated %: C, 49.69; H, 3.21; N, 26.74.

### 10‐Hydroxy‐2,4,9‐trimethyl‐7,8‐dihydropyrimido[5′′′,4′′′:5′′,6′′]pyrido[4′′,3′′:3′,4′]pyrazolo[1′,5′:1,2]pyrimidine‐1,3,8(2H,4H, 7H)‐trione (3 b)

Yield, 1.9 g 58 %; mp 273–276 °C; IR (KBr), ν, cm^−1^: 3250–3150 (OH, NH), 1630 (C=O). ^1^H NMR (DMSO‐*d_6_*), δ, ppm (*J*, Hz): 2.15 (3H, s, CH_3_), 2.35, 3.22 (6H, s, 2 NCH_3_), 7.98 (^1^H, s, HAr), 9.13 (^1^H, bs, NH), 10.12 (^1^H, bs, OH). ^13^C NMR (DMSO‐*d_6_*), δc, ppm: 19.6 (CH_3_), 29.3, 30.5 (2 N CH_3_), 91.2, 101.4, 125.7, 127.6, 129.4, 133.7, 147.6, 150.8 (11 C Ar), 158.5, 160.0 161.3 (3 C=O). Mass spectrum (EI, 70 eV), *m/z* (*I*_ref_, %): 328 (M^+^, 10), 280 (70), 312 (20), 261 (30), 106 (35), 77 (100). Found %: C, 51.39; H, 3.45; N, 25.42. C_14_H_12_N_6_O_4_ (328.29) Calculated C, 51.22; H, 3.68; N, 25.60.

### 9‐Ethyl‐10‐hydroxy‐2,4,‐dimethyl‐7,8‐dihydropyrimido[5′′′,4′′′:5′′,6′′]pyrido[4′′,3′′:3′,4′]pyrazolo[1′,5′:1,2]pyrimidine‐1,3,8(2H,4H, 7H)‐trione (3 c)

Yield, 2.4 g 70 %; mp 254–257 °C; IR (KBr), ν, cm^−1^: 3400–3180 (OH, NH), 1559 (C=O). ^1^H NMR (DMSO‐*d_6_*), δ, ppm (*J*, Hz): 1.11 (3H, t, *J=*6.95 Hz, CH_3_CH_2_), 2.23 (3H, s, CH_3_CH_2_), 2.65, 3.11 (6H, s, 2 NCH_3_), 7.22 (1H, s, HAr), 9.23 (1H, bs, NH), 12.08 (1H, bs, OH). ^13^C NMR (DMSO‐*d_6_*), δc, ppm: 15.6 (CH_2_CH_3_), 28.3, 29.5 (2 N−CH_3_), 87.2, 99.4, 110.7, 125.6, 127.4, 131.7, 145.6, 150.3, 150.9 (12 C Ar), 155.5, 156.0 159.8 (3 C=O). Mass spectrum (EI, 70 eV), *m/z* (*I*_ref_, %): 342 (M^+^, 3), 297 (5), 229 (25), 187 (100). Found %: C, 52.49; H, 4.05; N, 24.42. C_15_H_14_N_6_O_4_ (342.31) Calculated C, 52.63; H, 4.12; N, 24.55.

### 9‐n‐Butyl‐10‐Hydroxy‐2,4‐dimethyl‐7,8‐dihydropyrimido[5′′′,4′′′:5′′,6′′]pyrido[4′′,3′′:3′,4′]pyrazolo[1′,5′:1,2]pyrimidine‐1,3,8(2H,4H, 7H)‐trione (3 d)

Yield, 2.8 g 76 %; mp 254–257 °C; IR (KBr), ν, cm^−1^: 3310–3280 (OH, NH), 1554 (C=O). ^1^H NMR (DMSO‐*d_6_*), δ, ppm (*J*, Hz): 0.98 (3H, t, *J*=6.86 Hz, CH_3_CH_2_CH_2_CH_2_−), 2.55, 2.89 (6H, s, 2 NCH_3_), 3.22–4.45 (6H, m, CH_3_CH_2_CH_2_CH_2_−), 7.98 (1H, s, HAr), 9.76 (1H, bs, NH), 10.22 (1H, bs, OH). ^13^C NMR (DMSO‐*d_6_*), δc, ppm: 12.6, 15.7, 28.3, 29.5, 29.9, 33.2 (6 C‐aliph), 99.7, 111.7, 124.6, 125.8, 129.7, 144.6, 150.0, 152.9 (12 C Ar), 157.5, 158.5 165.8 (3 C=O). Mass spectrum (EI, 70 eV), *m/z* (*I*_ref_, %): 370 (M^+^, 3), 239 (35), 187 (100). Found %: C, 55.08; H, 4.63; N, 22.52. C_17_H_18_N_6_O_4_ (370.37) Calculated: C, 55.13; H, 4.90; N, 22.69.

### 10‐Hydroxy‐2,4,‐dimethyl‐9‐phenyl‐7,8‐dihydropyrimido[5′′′,4′′′:5′′,6′′]pyrido[4′′,3′′:3′,4′]pyrazolo[1′,5′:1,2]pyrimidine‐1,3,8(2H,4H, 7H)‐trione (3 e)

Yield, 2.7 g 69 %; mp 305–308 °C; IR (KBr), ν, cm^−1^: 3270–3100 (OH, NH), 1660 (C=O). ^1^H NMR (DMSO‐*d_6_*), δ, ppm (*J*, Hz): 2.15 (3H, s, CH_3_), 2.43, 3.18 (6H, s, 2 NCH_3_), 7.05–822 (5H, m, Ph), 8.33 (1H, s, HAr), 8.78 (1H, bs, NH), 13.12 (1H, bs, OH). ^13^C NMR (DMSO‐*d_6_*), δc, ppm: 27.3, 28.5 (2 N CH_3_), 81.2, 99.4, 111.6, 113.5, 114.8, 124.7, 126.6, 128.2 (2 C), 128.5 (2 C), 129.7, 133.6, 137.0, 145.6, 151.8 (16 C Ar), 156.2, 157.3 163.4 (3 C=O). Mass spectrum (EI, 70 eV), *m/z* (*I*_ref_, %): 390 (M^+^, 3), 335 (10), 175 (20), 119 (70), 77 (100). Found %: C, 58.29; H, 3.45; N, 21.32. C_19_H_14_N_6_O_4_ (390.36). Calculated: C, 58.46; H, 3.62; N, 21.53.

### 9‐(2‐Hydroxyethyl)‐10‐Hydroxy‐2,4,8‐trimethyl‐pyrimido[5′′′,4′′′:5′′,6′′]pyrido[4′′,3′′:3′,4′]pyrazolo[1′,5′:1,2]pyrimidine‐1,3,(2H,4H)‐dione (4)

To a solution of aminopyrazole 4 (3.7 g, 15 mmol) in glacial acetic acid (20 ml), 3‐acetyldihydrofuran‐2(3H)‐one (2.6 g, 20 mmol) was added. The mixture was refluxed for 5 h. until complete the reaction (tlc). After, cooling the precipitate was filtered off, to get the yellow powder product, which recrystallized from AcOH to obtain tetracyclic derivative 4. Yield 3.7 g 70 %, mp 315–318 °C. IR (KBr), ν, cm^−1^: 3230–3150 (OH, NH), 1630, 1644, 1688 (3 C=O). ^1^H NMR (DMSO‐*d_6_*), δ, ppm (*J*, Hz): 2.11 (3H, s, CH_3_), 2.34, 2.89 (6H, s, 2 NCH_3_), 3.55 (1H, bs, −CH_2_OH), 4.22–4.45 (4H, m, OHCH_2_CH_2_−), 8.22 (1H, s, HAr), 9.28 (1H, bs, NH). ^13^C NMR (DMSO‐*d_6_*), δc, ppm: 22.6, 25.7, 31.4, 34.7 (4‐C‐aliph), 89.5, 101.7, 113.5, 124.6, 124.8, 127.7, 139.6, 140.3, 149.4 (13 C−Ar), 153.5, 156.5 161.8 (3 CO). Mass spectrum (EI, 70 eV), *m/z* (*I*_ref_, %): 355 (M^+^ −1, 20), 301 (55), 220 (50), 170 (100). Found %: C, 53.64; H, 4.43; N, 23.42. C_16_H_16_N_6_O_4_ (356.34) Calculated: C, 53.93; H, 4.53; N, 23.58.

### 9‐(2‐Chloroethyl)‐10‐chloro‐2,4,8‐trimethyl‐pyrimido[5′′′,4′′′:5′′,6′′]pyrido[4′′,3′′:3′,4′]pyrazolo[1′,5′:1,2]pyrimidine‐1,3,(2H,4H)‐dione (5)

To a solution of dihdroxypyrimidine 6 (10.7 g, 30 mmol) in dry pyridine (15 ml), phosphorous oxychloride (6.8 g, 45 mmol) was added. The mixture was heated under reflux for 7 h. until obtained the clear reaction solution, then cool, and poured into ice portion by portion with steering. The colorless dichloro product was collected by filtration washed with water and dried. Yield 8.4 g 71 %, mp 290–293 °C. IR (KBr), ν, cm^−1^: 3150–3050 (CH Ar), 1658, 1690 (2 C=O). ^1^H NMR (CDCl_3_), δ, ppm (*J*, Hz): 2.14, 2.32, 2.55 (9H, 3 s, CH_3_, 2 NCH_3_), 2.98 (2H, t, *J*=6.95 Hz, −CH_2_CH_2_Cl), 4.22 (2H, t, *J*=7.05 Hz, CH_2_CH_2_Cl), 7.88 (1H, s, HAr). ^13^C NMR (DMSO‐*d_6_*), δc, ppm: 14.4, 23.7, 29.4, 33.7, 41.8 (5‐C‐aliph), 118.5, 119.7, 133.5, 141.6, 145.8, 147.7, 149.6, 151.3, 157.5, (14 C Ar), 159.5 168.8 (2 C=O). Mass spectrum (EI, 70 eV), *m/z* (*I*_ref_, %): 394 (M^+^+1, 2), 393 (M^+^, 5), 234 (100). Found %: C, 48.64; H, 3.48; N, 24.15. C_16_H_14_Cl_2_N_6_O_2_ (393.23) Calculated: C, 48.87; H, 3.59; N, 21.37.

### General Procedure for the Synthesis Trihydropyrimido[5′′′,4′′′:5′′,6′′]pyrido[4′′,3′′:3′,4′]pyrazolo[1′,5′:1,2]pyrimido[4,5‐c] pyrrolidine‐1,3(2H,4H)‐dione (6 a–d)

To a mixture of dichloride derivative **5** (0.8 g, 2 mmol) and an appropriate primary amine (4 mmol), in absolute ethanol (20 ml), 3 ml of triethyl amin was added in one portion. The reaction mixture was refluxed with stirring for 4–8 h until complete the reaction (tlc). Then cool, concentrated the solvent in *vacuo*, and the residue was treated with ether to get the colorless product which collected by filtration to give compounds **6 a**–**d** in reasonable yield.

### 2,4, 8,11‐Tetramethyl‐9,10,11‐Trihydropyrimido[5′′′,4′′′:5′′,6′′]pyrido[4′′,3′′:3′,4′]pyrazolo[1′,5′:1,2]pyrimido[4,5‐c] pyrrolidine‐1,3(2H,4H)‐dione (6 a)

Yield 0.5 g 71 %, mp 272–275 °C. IR (KBr), ν, cm^−1^: 3100–3050 (CH Ar), 2998–2877 (CH aliph), 1668, 1676 (2 C=O). ^1^H NMR (DMSO‐*d_6_*), δ, ppm (*J*, Hz): 2.14, 2.32, 2.55, 3.21 (12H, 4 s, CH_3_, 3 NCH_3_), 4.23 (2H, t, *J=*6.56 Hz, CH_2_ pyrrolidine), 4.43 (2H, t, *J=*7.44 Hz, CH_2_ pyrroldine), 8.41 (1H, s, HAr). ^13^C NMR (DMSO‐*d_6_*), δc, ppm: 16.3, 22.8, 27.4, 32.7, 39.8 (5‐C‐aliph), 117.4, 118.4, 129.5, 134.6, 138.8, 141.7, 143.6, 147.3, 149.8, 149.9 (10 C−Ar), 159.5 168.8 (2 C=O). Mass spectrum (EI, 70 eV), *m/z* (*I*_ref_, %): 351 (M^+^, 2), 239 (30). 187 (70), 140 (100). Found %: C, 58.04; H, 4.68; N, 27.75. C_17_H_17_N_7_O_2_ (351.37) Calculated: C, 58.11; H, 4.88; N, 27.90.

### 11‐Ethyl‐2,4,8‐trimethyl‐9,10,11‐trihydropyrimido[5′′′,4′′′:5′′,6′′]pyrido[4′′,3′′:3′,4′]pyrazolo[1′,5′:1,2]pyrimido[4,5‐cpyrrolidine]‐1,3(2H,4H)‐dione (6 b)

Yield 0.52 g 70 %, mp 250–254 °C. IR (KBr), ν, cm^−1^: 3150–3100 (CH Ar), 2995–2855 (CH aliph), 1670, 1673 (2‐CO‐lactam). ^1^H NMR (DMSO‐*d_6_*), δ, ppm (*J*, Hz): 1.12 (3H, t, *J=*6.96 Hz, CH_3_CH_2_), 2.17, 2.22, 2.57, 3.24 (11H, m, CH_3_, 2 NCH_3_, CH_2_CH_3_), 4.26 (2H, t, *J=*6.56 Hz, CH_2_ pyrrolidine), 4.36 (2H, t, *J=*7.44 Hz, CH_2_ pyrroldine), 8.32 (1H, s, HAr). ^13^C NMR (DMSO‐*d_6_*), δc, ppm: 13.3, 16.8, 25.2, 27.7, 28.8, 30.3, 39.4 (CH_3_CH_2_, CH_2_CH_2_, CH_3_, 2 N−CH_3_), 115.2, 119.4, 122.5, 124.6, 137.4, 139.7, 141.6, 146.3, 148.8 (9 C Ar), 161.5 164.6 (2 C=O). Mass spectrum (EI, 70 eV), *m/z* (*I*_ref_, %): 365 (M^+^, 2), 364 (M^+^−1, 4), 201 (50), 147 (100). Found %: C, 59.10; H, 5.12; N, 26.65. C_18_H_19_N_7_O_2_ (365.40) Calculated: C, 59.17; H, 5.24; N, 26.83.

### 11‐(2‐Hydroxyethyl)‐2,4,8‐trimethyl‐9,10,11‐trihydropyrimido[5′′′,4′′′:5′′,6′′]pyrido[4′′,3′′:3′,4′]pyrazolo[1′,5′:1,2]pyrimido[4,5‐c] pyrrolidine‐1,3(2H,4H)‐dione (6 c)

Yield 0.50 g 63 %, mp 277–280 °C. IR (KBr), ν, cm^−1^: 3360 (−OH−), 3130–3100 (CH Ar), 1651, 1690 (2‐CO‐lactam). ^1^H NMR (DMSO‐*d_6_*), δ, ppm (*J*, Hz): 2.22, 3.15, 3.24 (9H, m, CH_3_, 2 NCH_3_), 4.14–4.25 (7H, m, N−CH_2_CH_2_OH, 2‐CH_2_ pyrrolidine, OH), 4.47 (2H, t, *J=*7.44 Hz, N−CH_2_CH_2_OH), 8.78 (1H, s, HAr). ^13^C NMR (DMSO‐*d_6_*), δc, ppm: 17.6, 19.8, 20.2, 23.7, 24.6, 27.3, 29.4 (7‐C‐aliph), 113.2, 115.2, 117.5, 118.6, 122.4, 128.7, 131.6, 133.3, 135.8 (9 C Ar), 159.5 161.9 (2 CO). Mass spectrum (EI, 70 eV), *m/z* (*I*_ref_, %): 382 (M^+^+1, 20), 381 (M^+^, 60), 293 (100). Found %: C, 56.44; H, 4.89; N, 25.75. C_18_H_19_N_7_O_3_ (365.40) Calculated: C, 56.69; H, 5.02; N, 25.71.

### 2,4,8‐Trimethyl‐11‐phenyl‐9,10,11‐trihydropyrimido[5′′′,4′′′:5′′,6′′]pyrido[4′′,3′′:3′,4′]pyrazolo[1′,5′:1,2]pyrimido[4,5‐c] pyrrolidine‐1,3(2H,4H)‐dione (6 d)

Yield 0.62 g 74 %, mp above 300 °C. IR (KBr), ν, cm^−1^: 3170–3090 (CH Ar), 1663, 1708 (2 C=O). ^1^H NMR (DMSO‐*d_6_*), δ, ppm (*J*, Hz): 2.32, 3.33, 3.36 (9H, 3 s, CH_3_, 2 NCH_3_), 4.33 (2H, t, *J=*9.0 Hz, CH_2_ pyrrolidine), 4.46 (2H, t, *J=*9.0 Hz, CH2 pyrroldine), 6.38–6.77 (5H, m, Ph), 8.54 (1H, s, HAr). ^13^C NMR (DMSO‐*d_6_*), δc, ppm: 19.3, 20.8, 24.4, .32.5, 37, 39.8 (5‐C‐aliph), 112.4, 113.4, 121.5, 123.6, 137.8, 139.7, 140.5, 143.7, 144.6, 146.4, 147.3, 148.6, 149.8, 149.9 (14 C Ar), 165.8 172.8 (2 C=O). Mass spectrum (EI, 70 eV), *m/z* (*I*_ref_, %): 413 (M^+^, 55), 375 (30). 220 (70), 170 (100). Found %: C, 63.74; H, 4.48; N, 23.55. C_22_H_19_N_7_O_2_ (413.44) Calculated: C, 63.91; H, 4.63; N, 23.72.

### 5,9,11‐Trimethyl‐1‐phenyl‐1,2,3,4‐tetrahydropyrimido[5′′′,4′′′:5′′,6′′]pyrido[4′′,3′′:3′,4′]pyrazolo[1′,5′:1,2]pyrimido[4,5‐c]pyridazine‐10,12(9H,11H)‐dione (7)

To a suspension solution of absolute ethanol containing Na_2_CO_4_ (1.3 g, 12 mmol), dichloride derivative **5** (1.2 g, 3 mmol), and phenyl hydrazine (0.7 g, 3 mmol) were added. The mixture was refluxed for 6 h (tlc). Concentrated the reaction in vacuum and the residue was dissolved in distillated water to get the solid crystals which was collected by filtration. Crystalized from methanol to obtain the pale yellow powder. Yield 0.72 g 58 %, mp 198–201 °C. IR (KBr), ν, cm^−1^:3400–3350 (−NH), 3100–3040 (CH Ar), 1667, 1700 (2 C=O). ^1^H NMR (DMSO‐*d_6_*), δ, ppm (*J*, Hz): 2.09, 3.31, 3.34 (9H, 3 s, CH_3_, 2 NCH_3_), 4.66 (2H, t, *J=*7.3 Hz, CH_2_ pyrrolidine), 5.08 (2H, t, *J=*7.3 Hz, CH_2_ pyrroldine), 6.26 6.44 (5H, m, Ph), 8.71 (1H, s, HAr), 10.55 (1H, bs, NH). ^13^C NMR (DMSO‐*d_6_*), δc, ppm: 18.0, 21.7, 23.0, .33.5, 40.8 (5‐C‐aliph), 111.2, 113.7, 120.3, 123.9, 125.8, 127.6, 137.5, 139.7, 140.8, 141.3, 142.6, 145.8, 146.4, 147.8, 148.3 (15 C−Ar), 164.2 167.5 (2 −CO). Mass spectrum (EI, 70 eV), *m/z* (*I*_ref_, %): 429 (M^+^+1, 20), 388 (5). 364 (10), 363 (100). Found %: C, 61.43; H, 4.58; N, 26.11. C_22_H_20_N_8_O_2_ (428.46) Calculated: C, 61.67; H, 4.71; N, 26.15.

### 2,4,8‐Trimethyl‐9,10,11‐trihydropyrimido[5′′′,4′′′:5′′,6′′]pyrido[4′′,3′′:3′,4′]pyrazolo[1′,5′:1,2]pyrimido[4,5‐c]thiophene‐1,3(2H,4H)‐dione (8)

To a solution of dichloro derivative **5** (0.8 g, 2 mmol) in 20 ml ethanol, thiourea (0.2 g, 2.6 mmol) was added. The mixture was heated under reflux for 4 h, then, the reaction concentrated under *vacuo* and the residue treated with K_2_CO_3_ solution (15 ml, 3 N). The resulting solution was extracted with CHCl_3_ (two times) and the extracted dried over anhydrous CaCl_2_. The chloroform was evaporated under reduced pressure and the residue was treated with ethanol (95 %) to obtain the pale yellow powder which recrystallized from ethanol. Yield 0.43 g 61 %, mp 252–255 °C. IR (KBr), ν, cm^−1^: 3060–3000 (CH Ar), 1650, 1690 (2‐CO−). ^1^H NMR (DMSO‐*d_6_*), δ, ppm (*J*, Hz): 2.14, 3.28, 3.36 (9H, 3 s, CH_3_, 3 NCH_3_), 3.77 (2H, t, *J*=8.5 Hz, CH_2_ pyrrolidine), 4.09 (2H, t, *J*=8.5 Hz, CH_2_ pyrroldine), 8.32 (1H, s, HAr). ^13^C NMR (DMSO‐*d_6_*), δc, ppm: 15.3, 17.8, 19.4, 33.7, 40.8 (5‐C‐aliph), 118.4, 129.5, 134.6, 138.8, 139.7, 142.6, 145.3, 149.8, 151.9 (9 C−Ar), 157.5 158.8 (2 C=O). Mass spectrum (EI, 70 eV), *m/z* (*I*_ref_, %): 354 (M^+^, 2), 351 (30). 145 (70), 117 (10), 90 (100). Found %: C, 54.16; H, 3.78; N, 23.66. C_16_H_14_N_6_O_2_S (354.39) Calculated: C, 54.23; H, 3.98; N, 23.71.

## Conclusions

Polyheterocyclic compounds containing four and five fused rings were prepared from the pyrazolo[3′,4′:4,5]pyrido[2,3‐d]pyrimidine as precursors. The preparation was accomplished by cyclocondensation reactions with malonates derivatives. But on the other hand, Dichloro derivatives undergo to nucleophilic cyclocondensation reactions, after its treatment with primary amines, phenyl hydrazine and/or thiourea, affording the polyherocyclic derivatives. The compounds have demonstrated potent antimicrobial activities against various microorganisms with sub‐minimal inhibitory concentrations. This study highlights the importance of rational drug design and synthetic strategies in developing novel compounds with potential therapeutic applications.

## Supplementary Materials

The supplementary material concerning the ^1^H‐NMR, ^13^C‐NMR and Mass spectra (A GC MS‐QP 1000 EX Mass Spectrometer was used to obtain mass spectra).

## Conflict of interests

The authors have no conflicts of interest to declare that are relevant to the content of this article.

1

## Supporting information

As a service to our authors and readers, this journal provides supporting information supplied by the authors. Such materials are peer reviewed and may be re‐organized for online delivery, but are not copy‐edited or typeset. Technical support issues arising from supporting information (other than missing files) should be addressed to the authors.

Supporting Information

## Data Availability

Data are submitted with the manuscript, as a supplementary files, also available upon requested.
