# Supplementary material for: Design and Synthesis of Polyheterocyclic Compounds Containing Pyrazolopyridopyrimidine Nucleus with Antimicrobial Activities
Source: ChemistryOpen. 2024 Apr 29;13(6):e202400070. doi: 10.1002/open.202400070 (PMC11164023; doi:10.1002/open.202400070)

ChemistryOpen

Supporting Information

Design and Synthesis of Polyheterocyclic Compounds Containing Pyrazolopyridopyrimidine Nucleus with Antimicrobial Activities

Farag A. El-Essawy* and Mohammad Ahmad Ahmad Odah

Design and Synthesis of Polyheterocyclic compounds containing Pyrazolopyridopyrimidine Nucleus with Antimicrobial Activities

Farag A. El-Essawy*(https://orcid.org/0000-0003-0683-325X) Mohammad Odah(https://orcid.org/0000-0003-1883-6992)

***email: f.elessawy@psau.edu.sa**

Supplementary materials

Including the MS, H, C-NMR spectral data: -

MS and ^{1}H NMR for Compound No. (2)

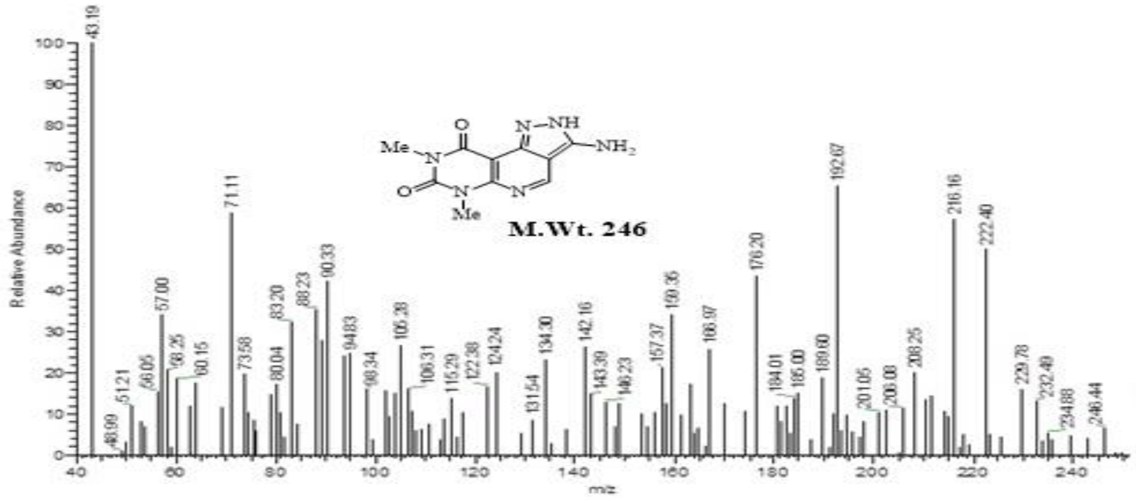

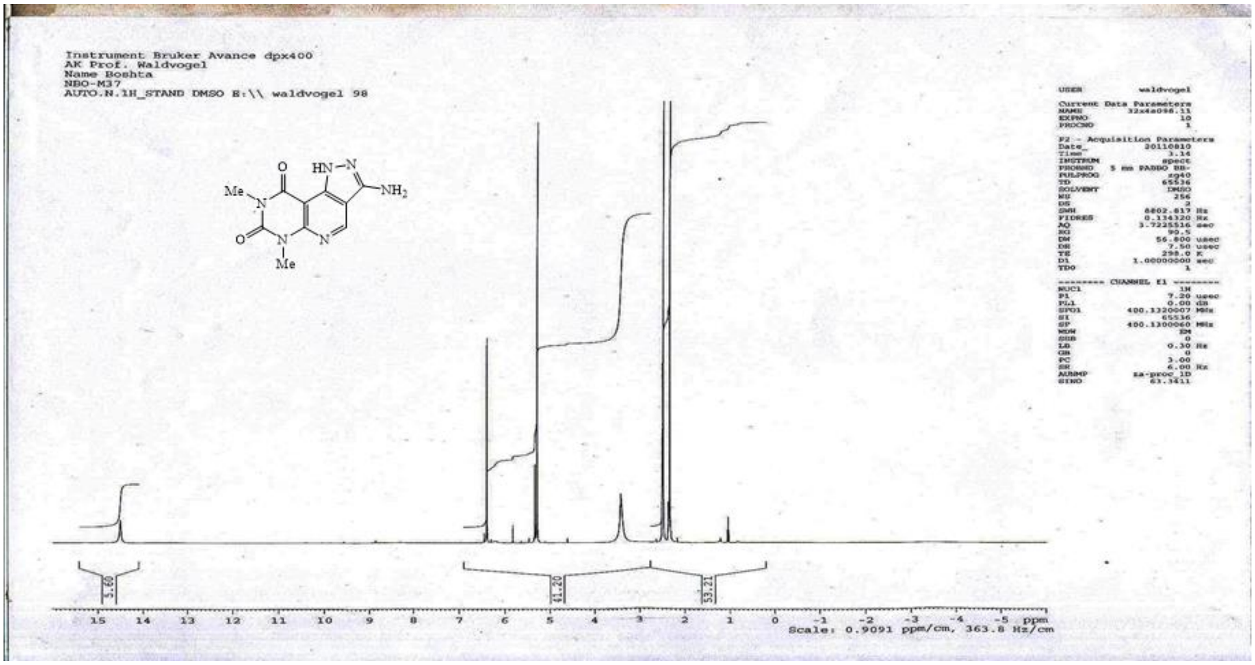

MS, ^{1}H NMR and ^{13}C for Compound No. (**3a**)

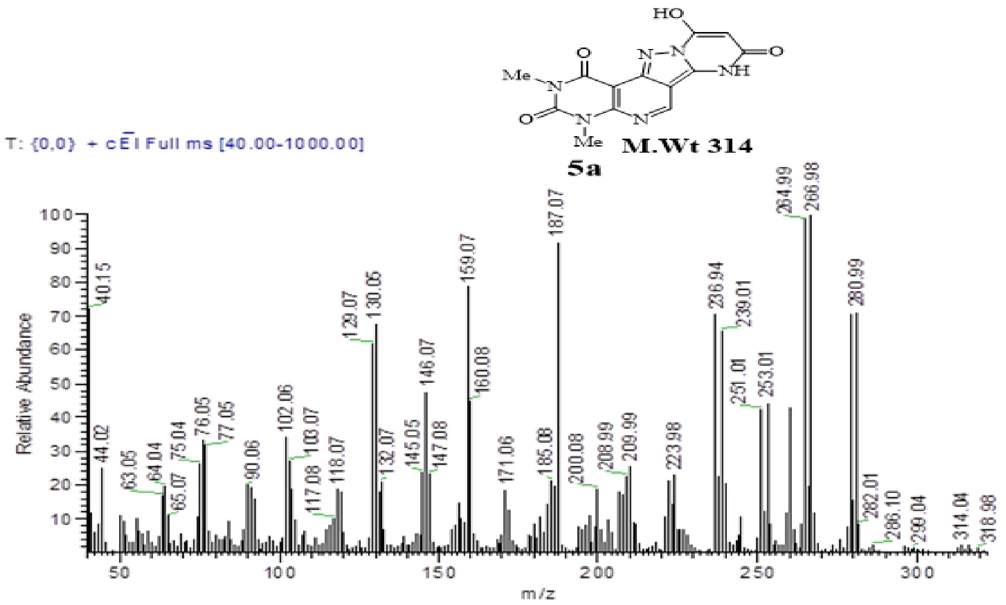

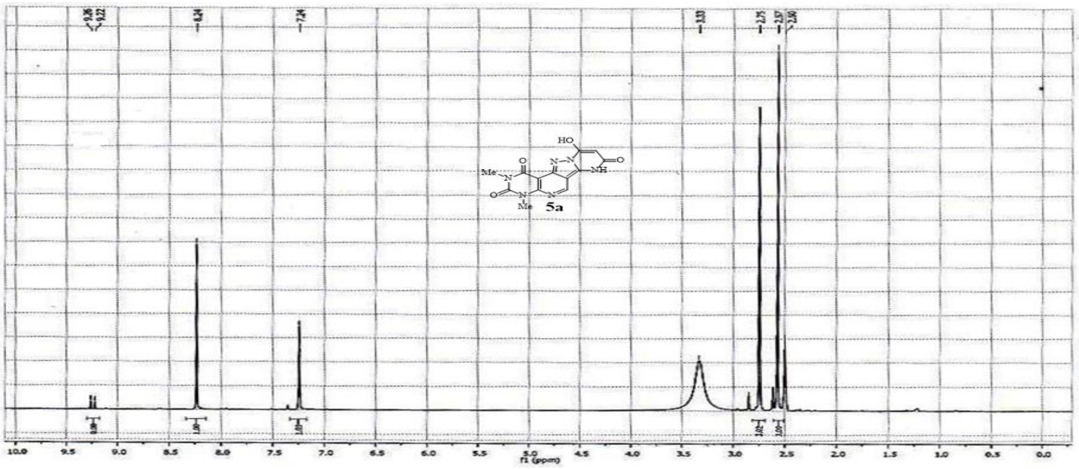

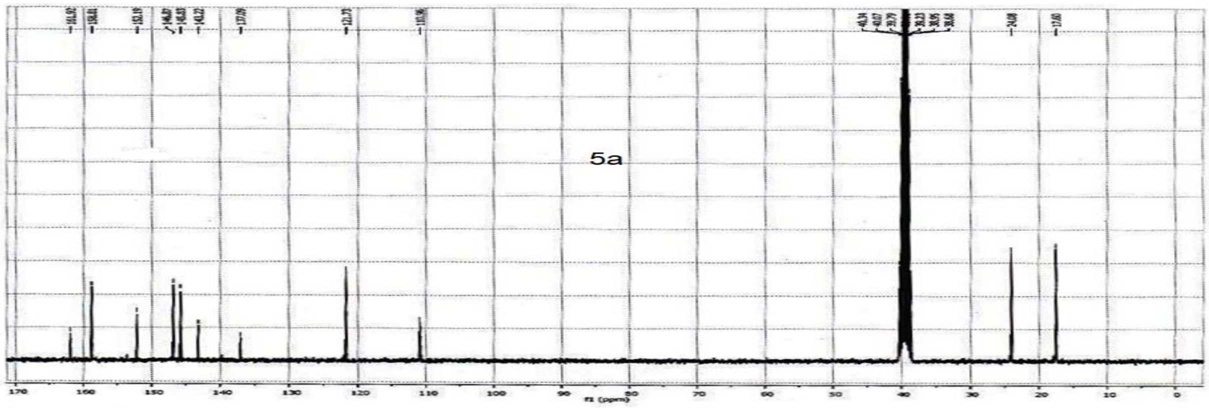

MS, ^{1}H NMR and ^{13}C for Compound No. (3b)

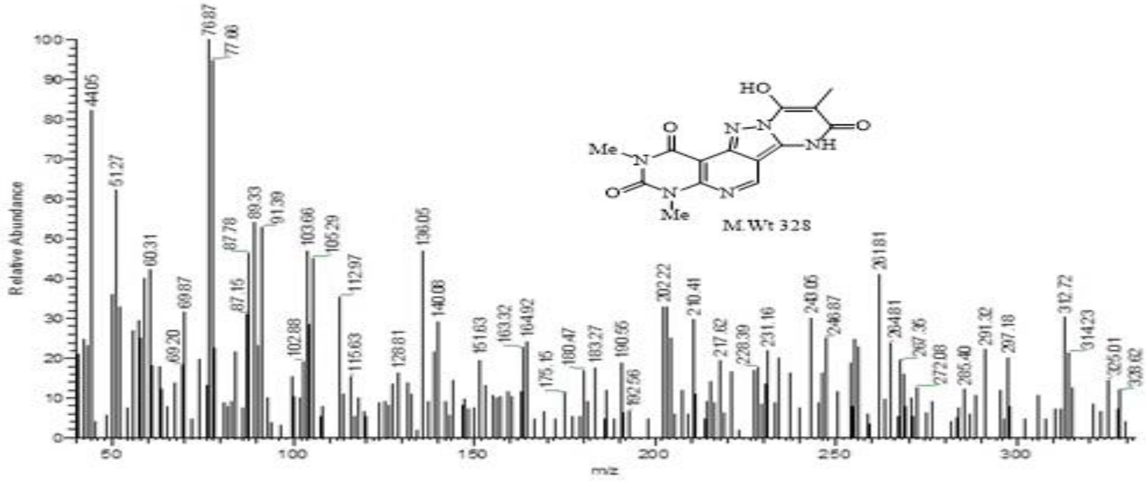

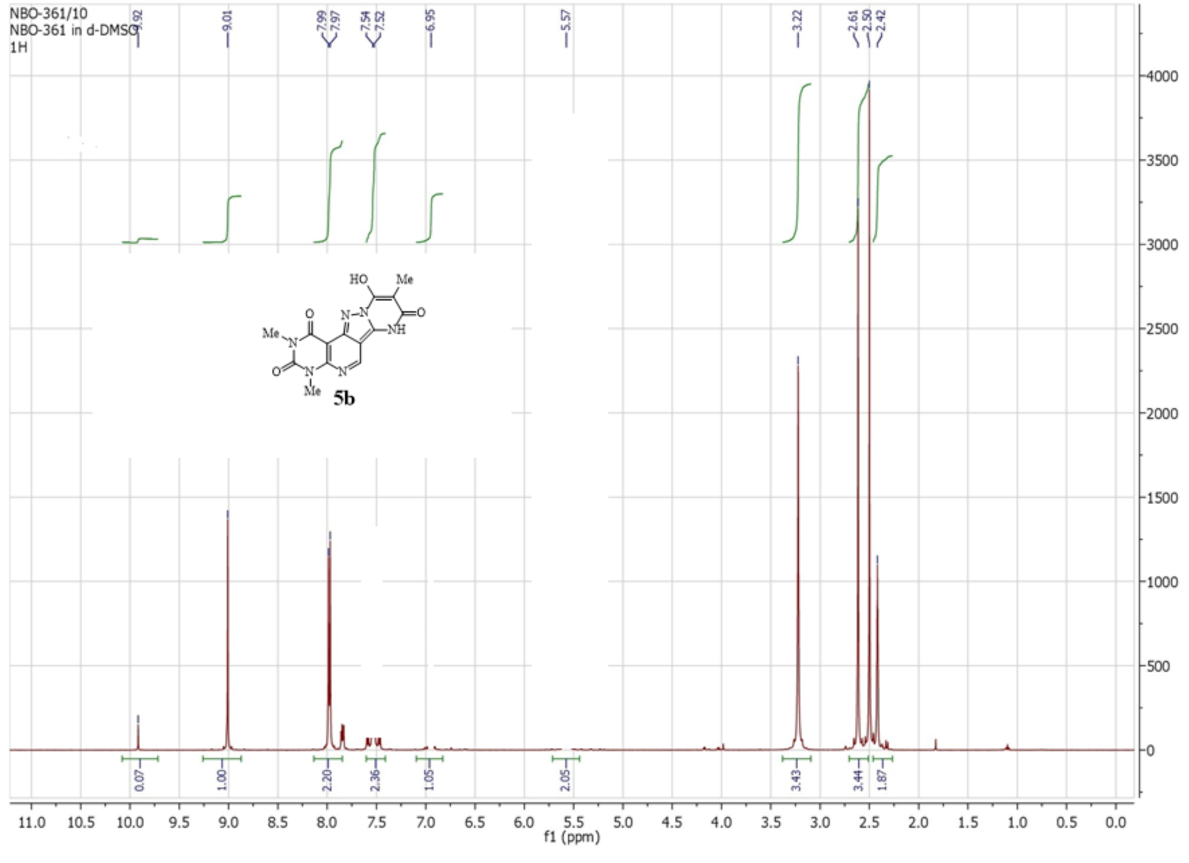

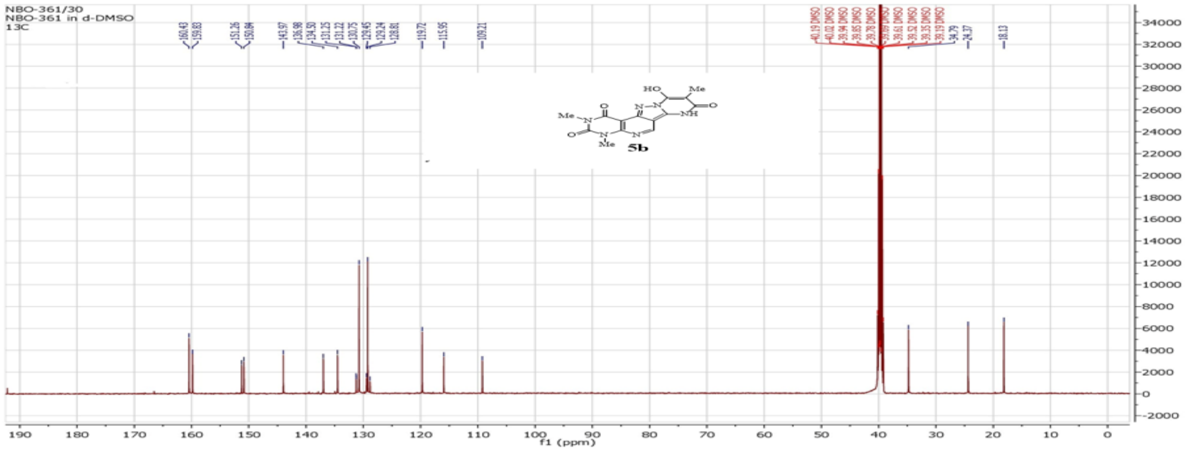

NBO-361/30
NBO-361 in d-DMSO
13C
160.43
159.83
151.26
150.84
143.97
136.98
134.50
131.25
131.22
130.75
129.45
129.24
128.81
119.72
115.95
109.21
40.19 DMSO
40.02 DMSO
39.94 DMSO
39.85 DMSO
39.78 DMSO
39.69 DMSO
39.61 DMSO
39.52 DMSO
39.35 DMSO
39.19 DMSO
34.79
24.37
18.13
HO
Me
NH
5b
f1 (ppm)

MS and H NMR for Compound 3c

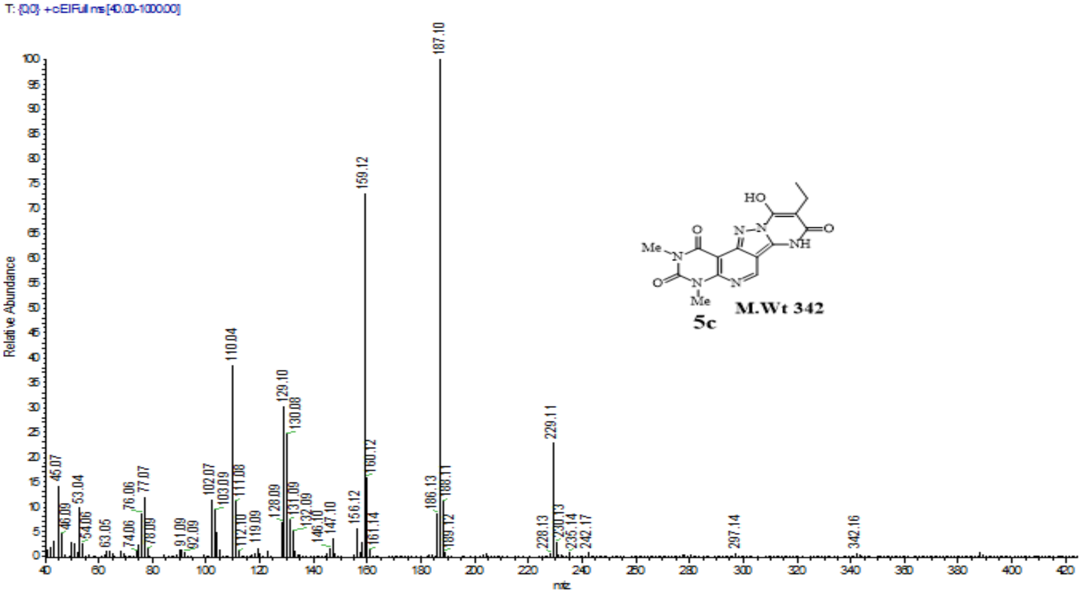

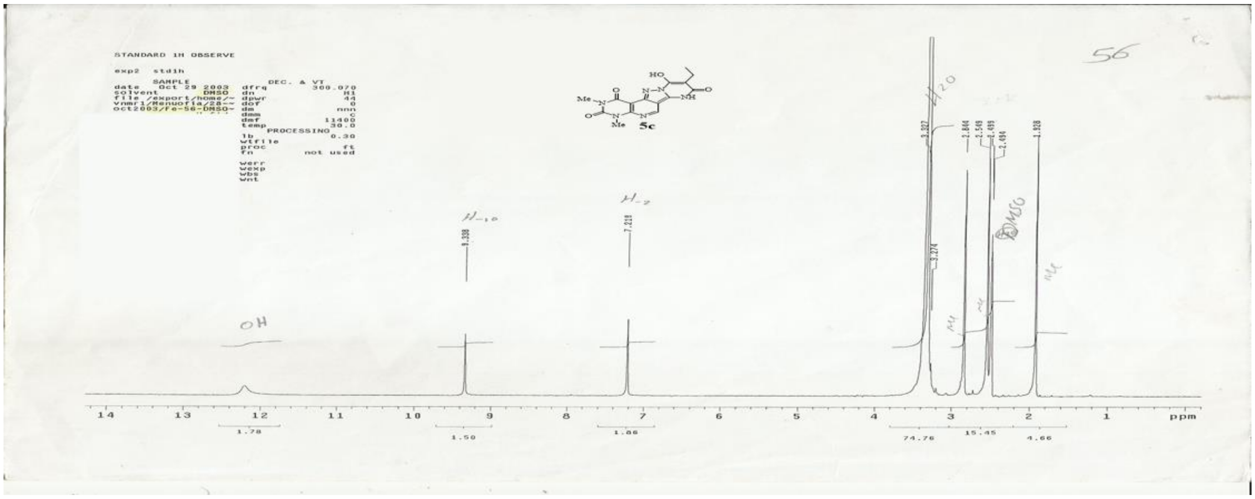

MS and H NMR for Compound (3d)

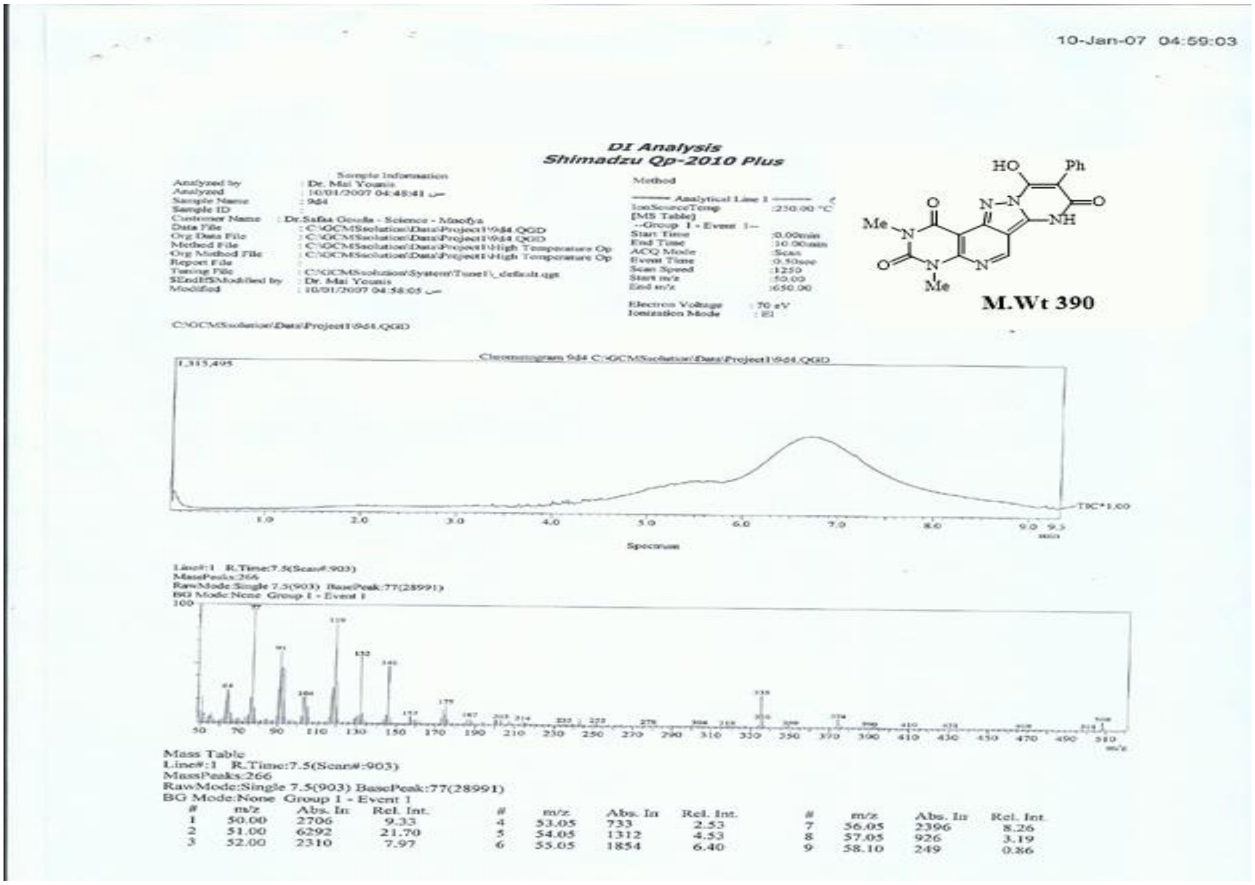

#	m/z	Abs. In	Rel. Int.	#	m/z	Abs. In	Rel. Int.	#	m/z	Abs. In	Rel. Int.
1	50.00	2706	9.33	4	53.05	733	2.53	7	56.05	2396	8.26
2	51.00	6292	21.70	5	54.05	1312	4.53	8	57.05	926	3.19
3	52.00	2310	7.97	6	55.05	1854	6.40	9	58.10	249	0.86

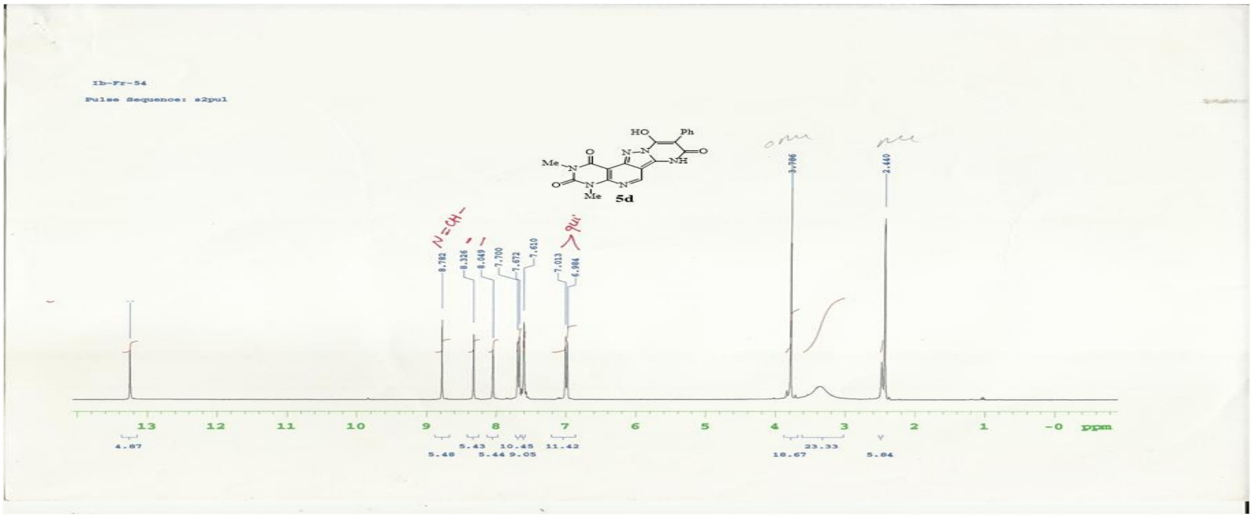

MS and H NMR for Compound No (3e)

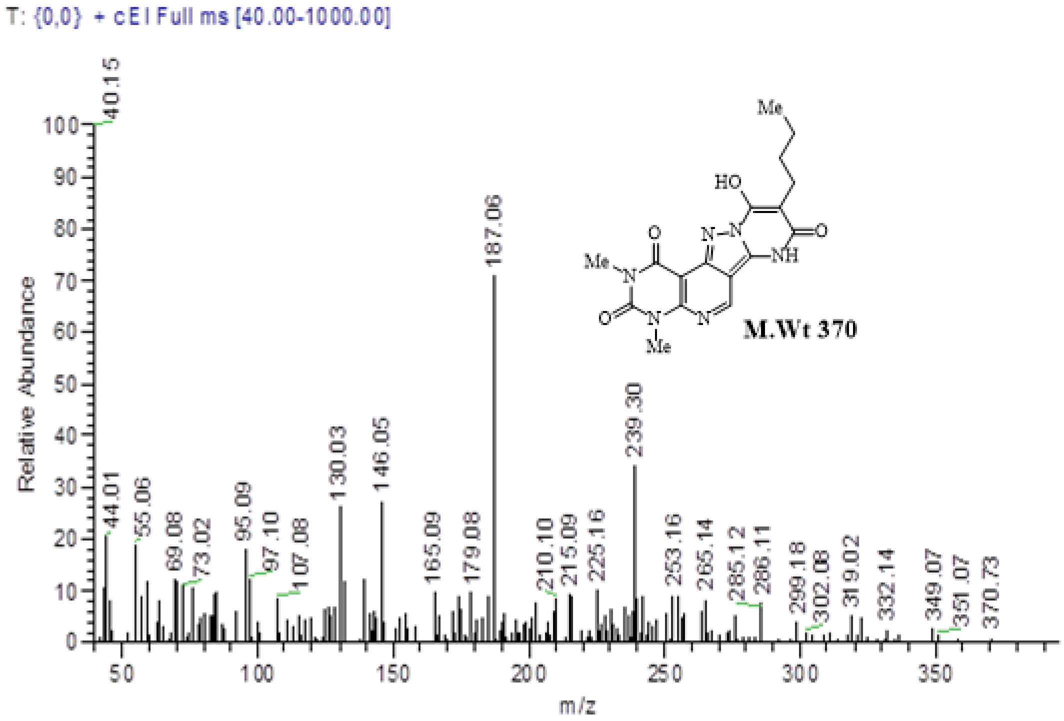

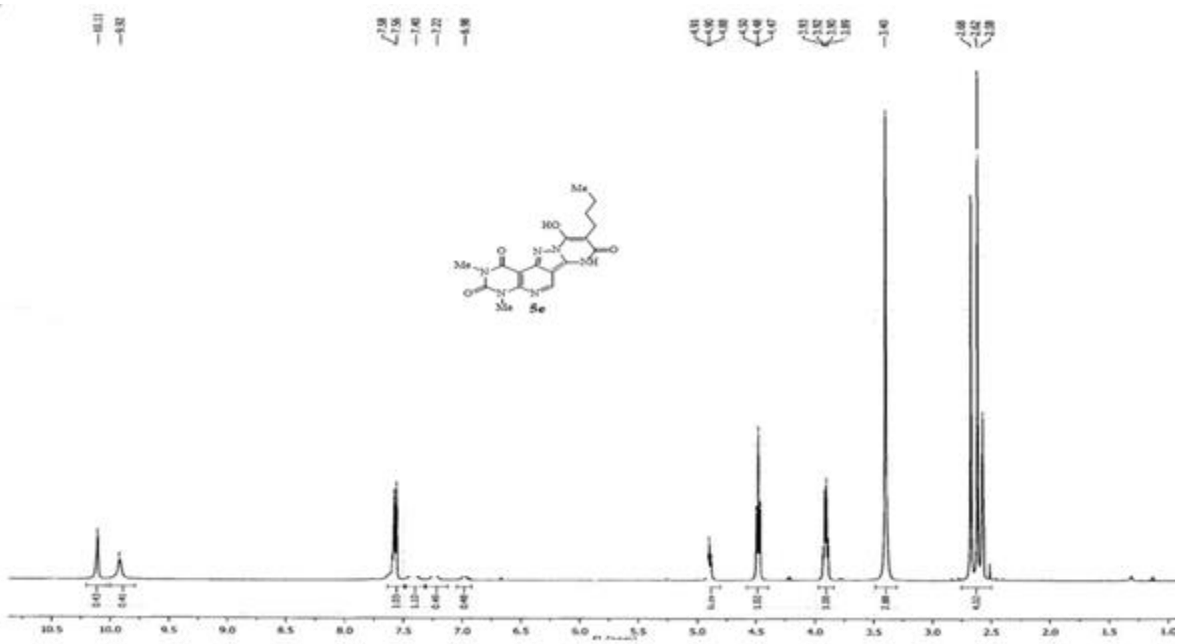

MS and H NMR for Compound (4)

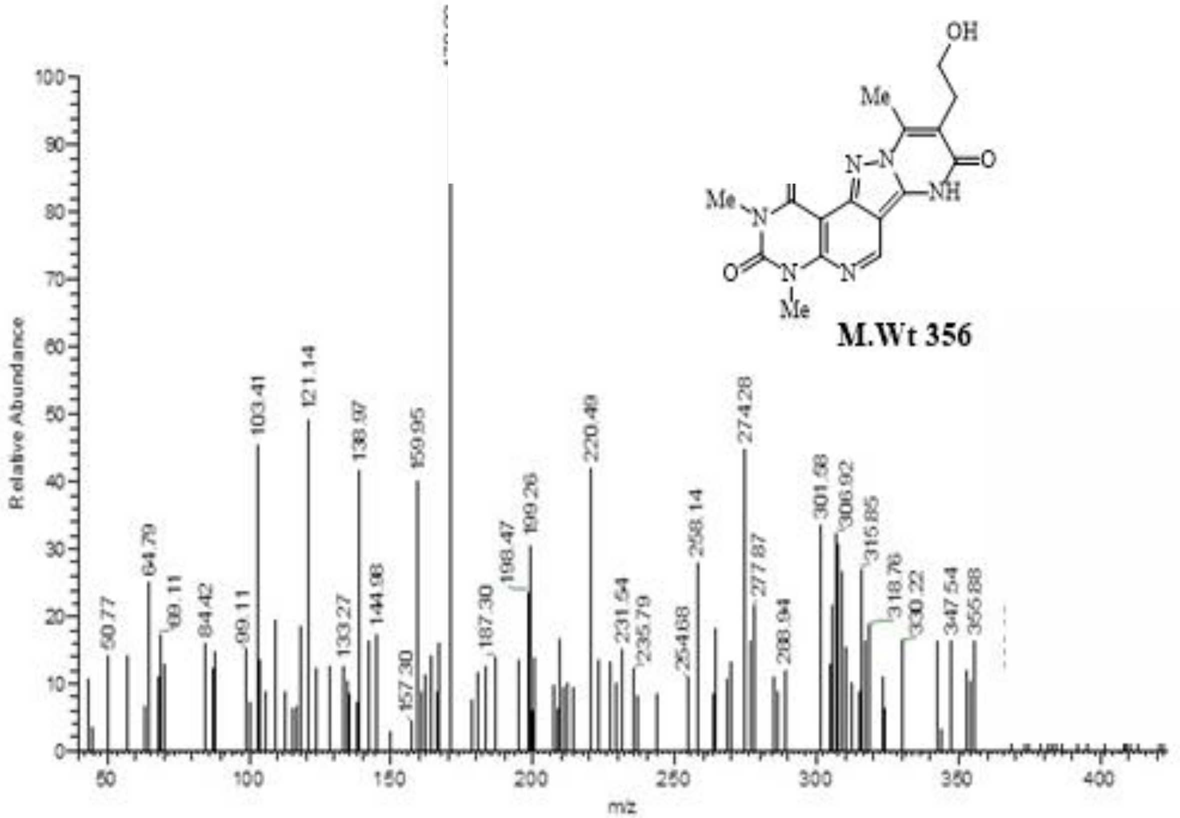

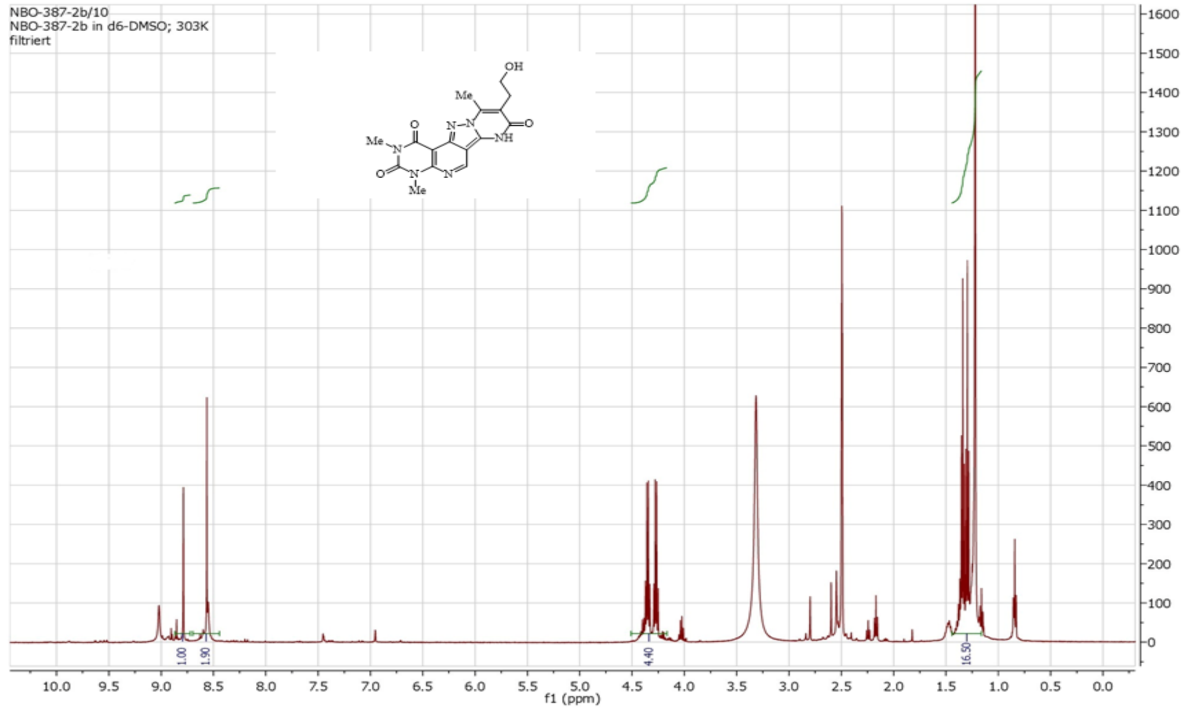

MS and ^{1}H NMR for Compound (5)

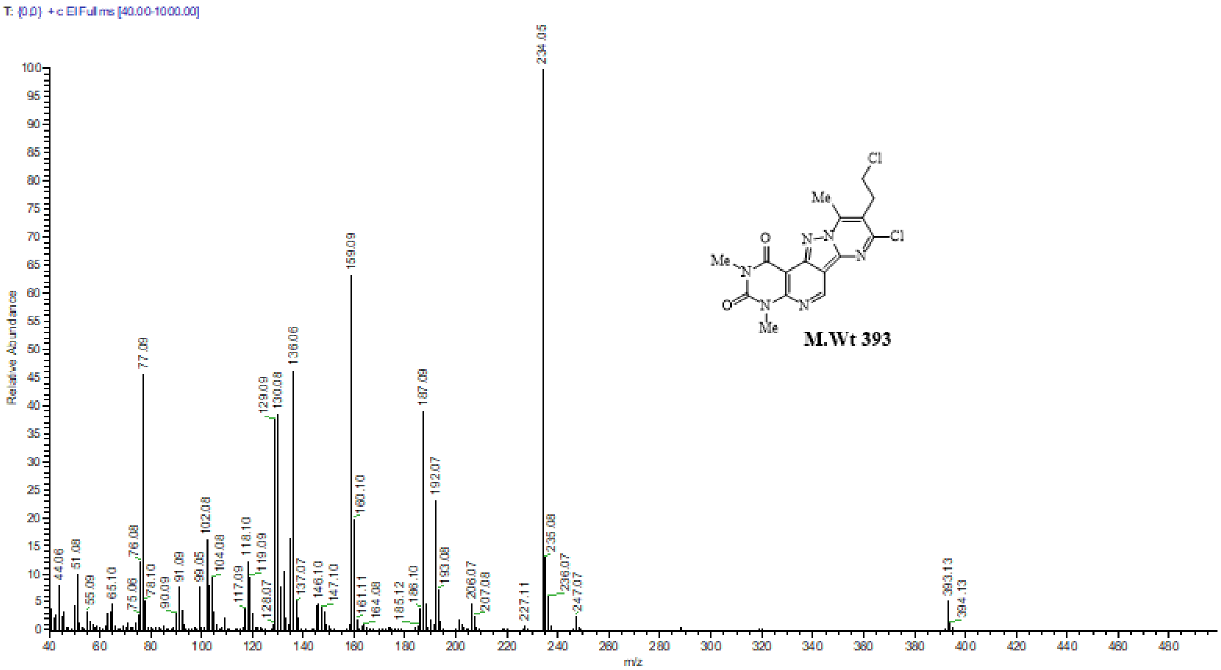

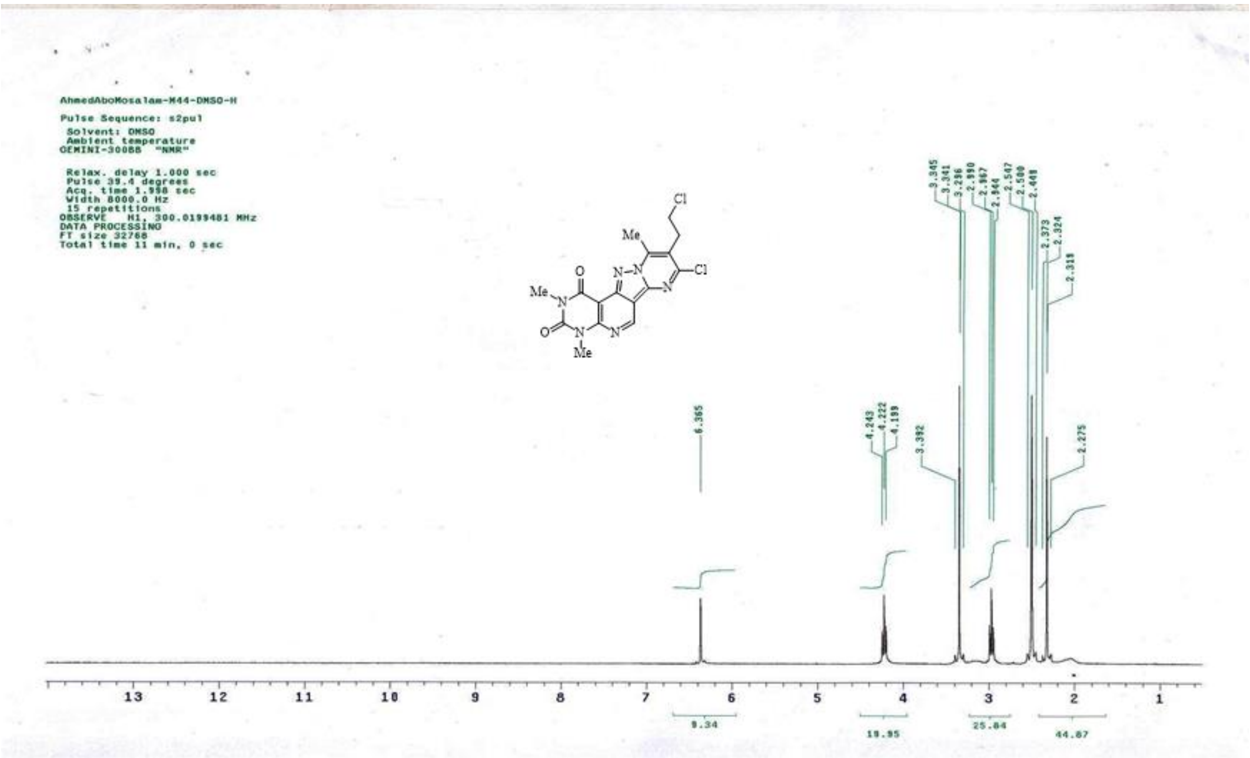

MS and ¹H NMR for Compound no (6a)

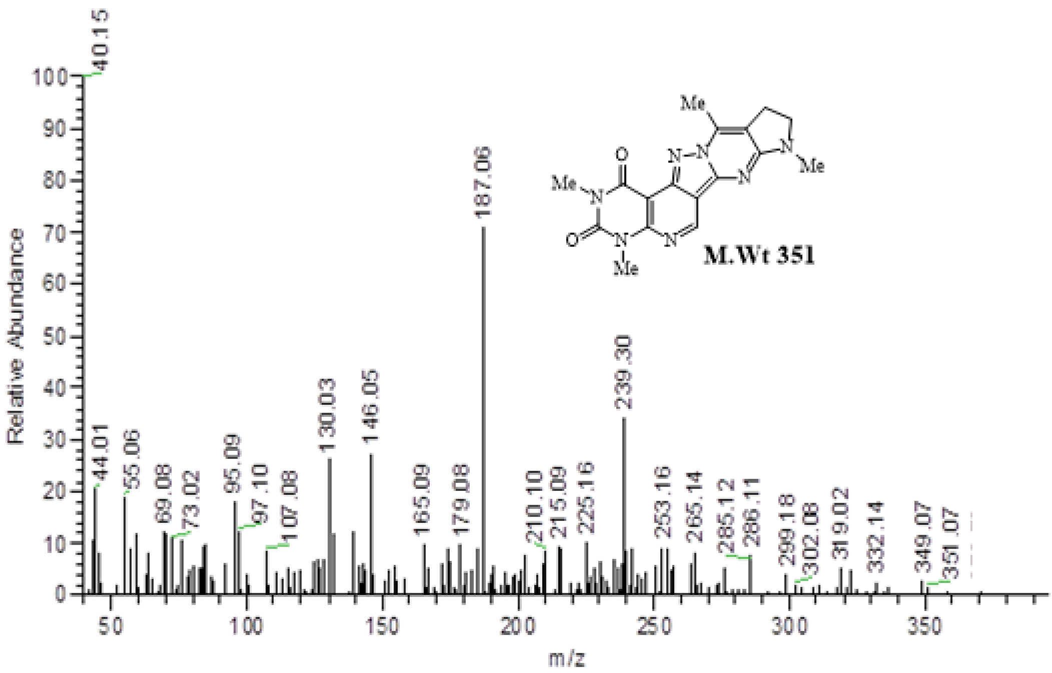

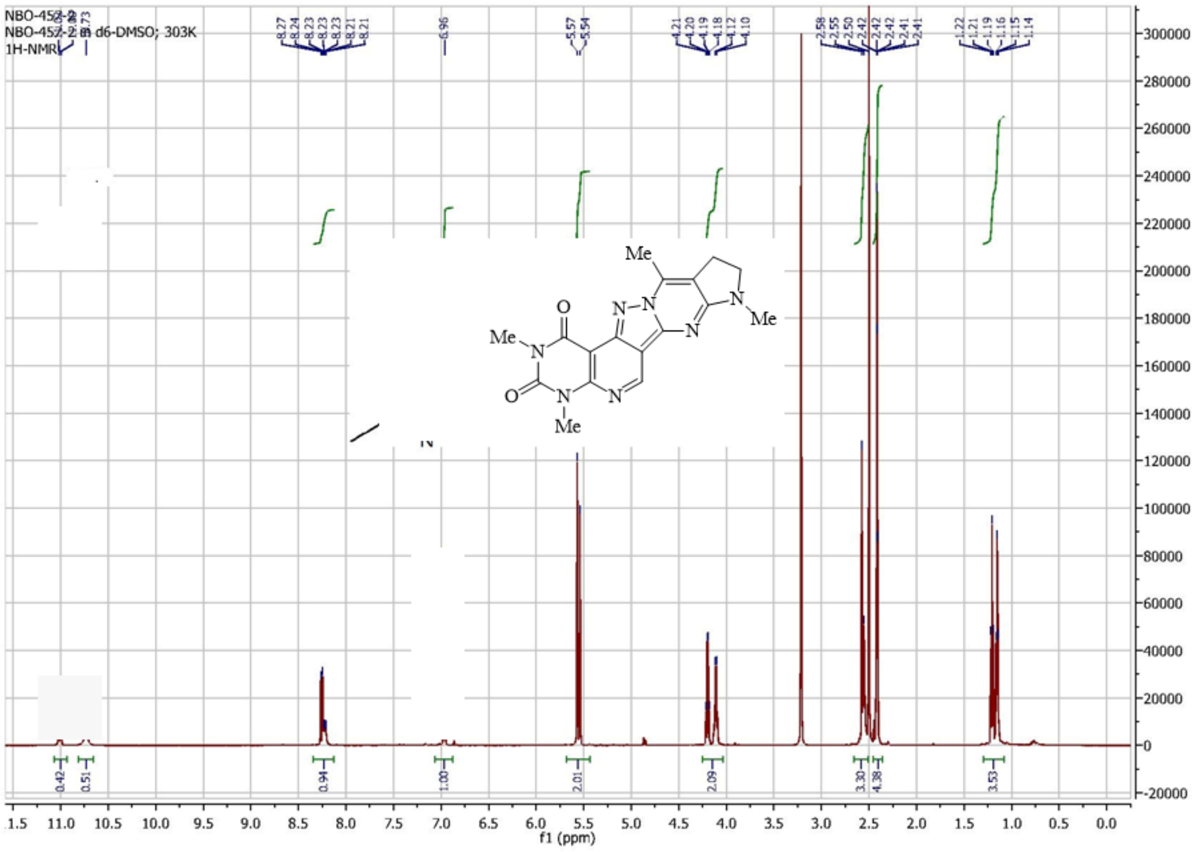

MS and ^{1}H NMR for Compound no (6b)

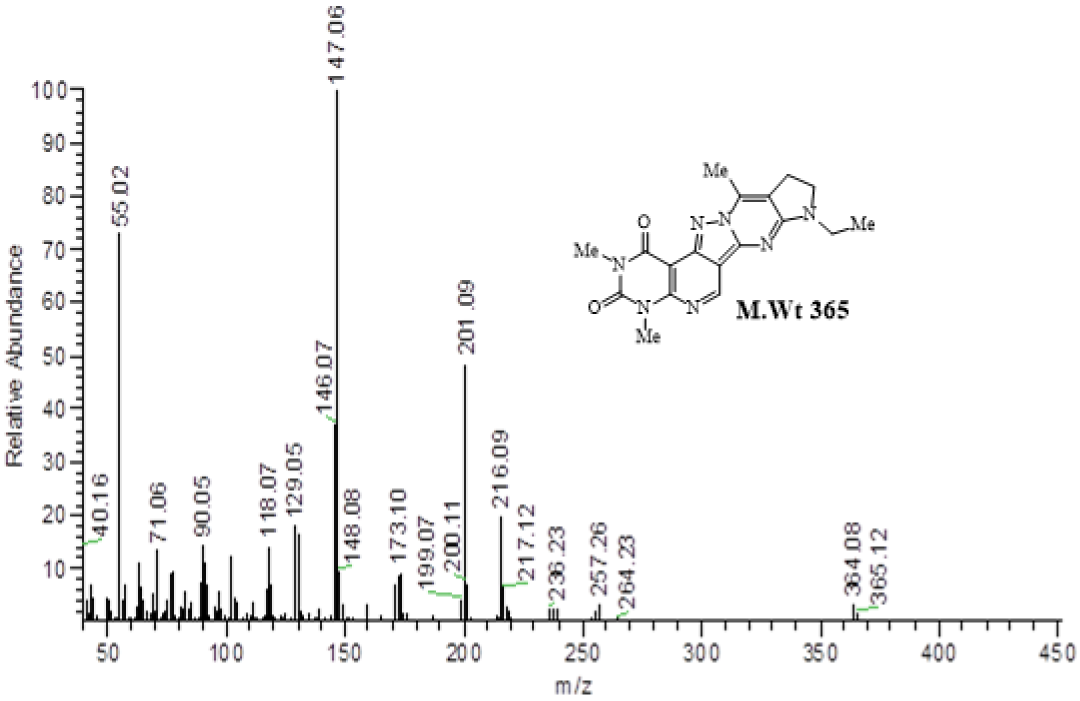

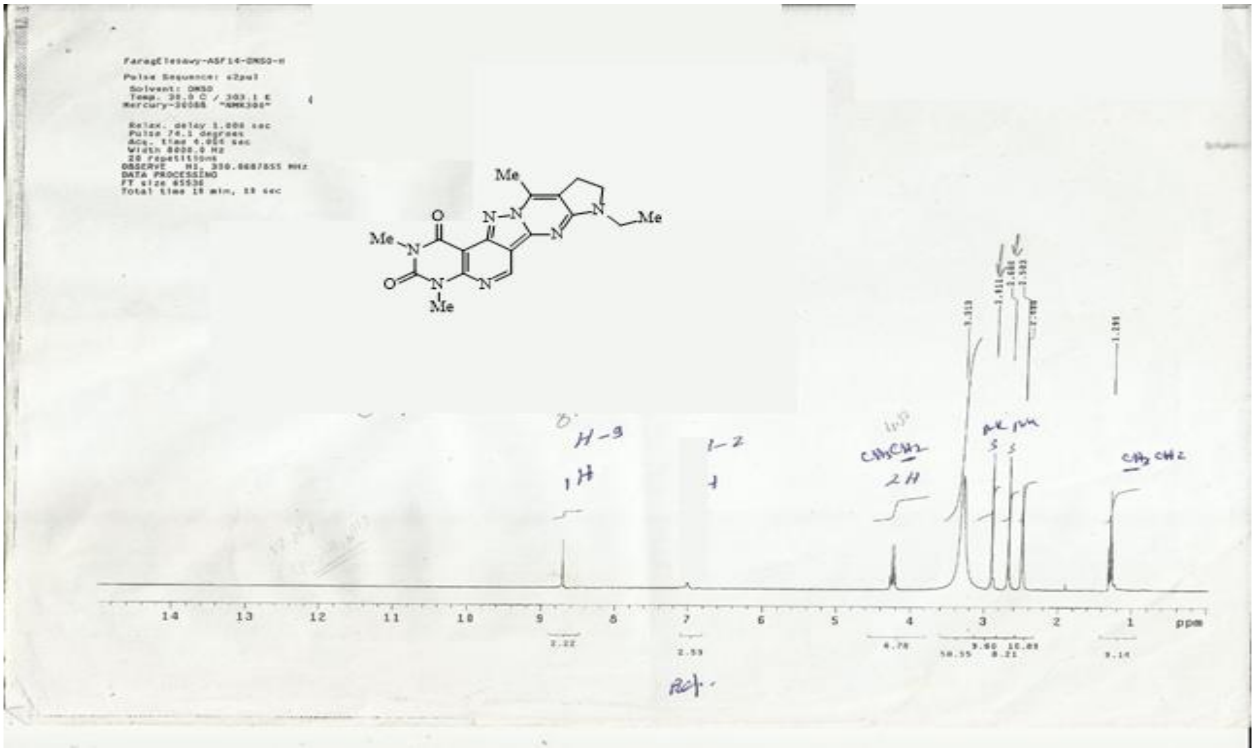

MS and ^{1}H NMR for Compound no (6c)

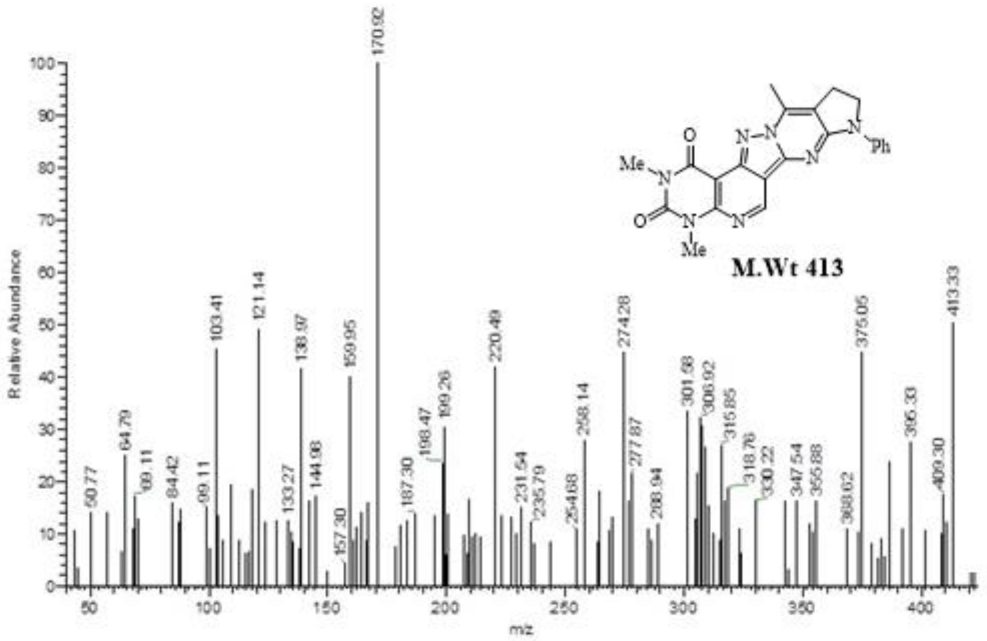

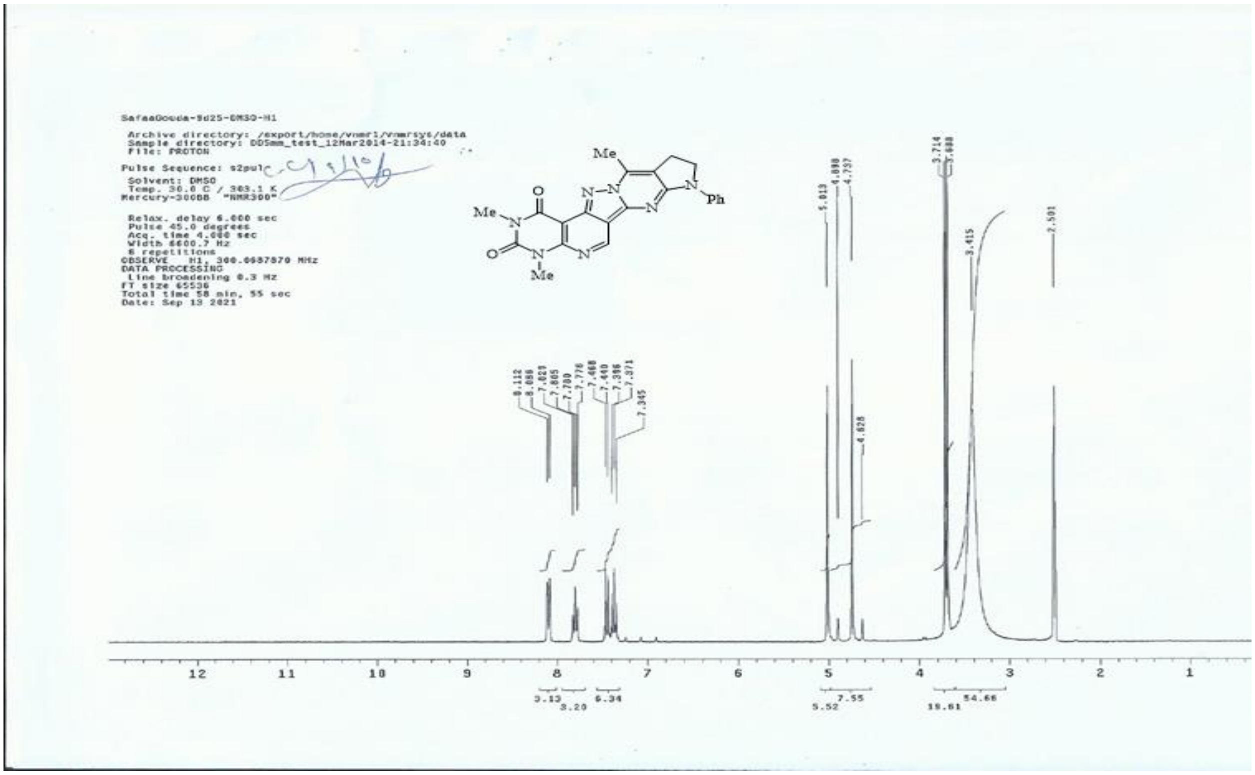

MS and ^{1}H NMR for Compound no (6d)

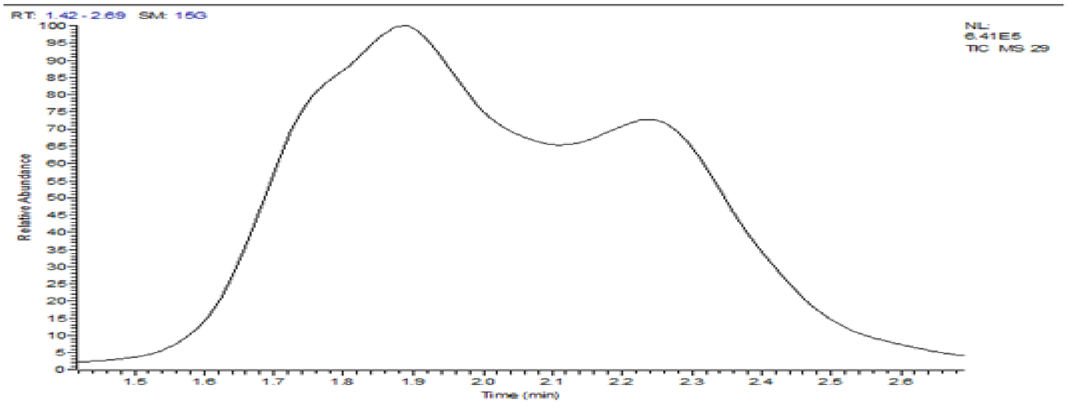

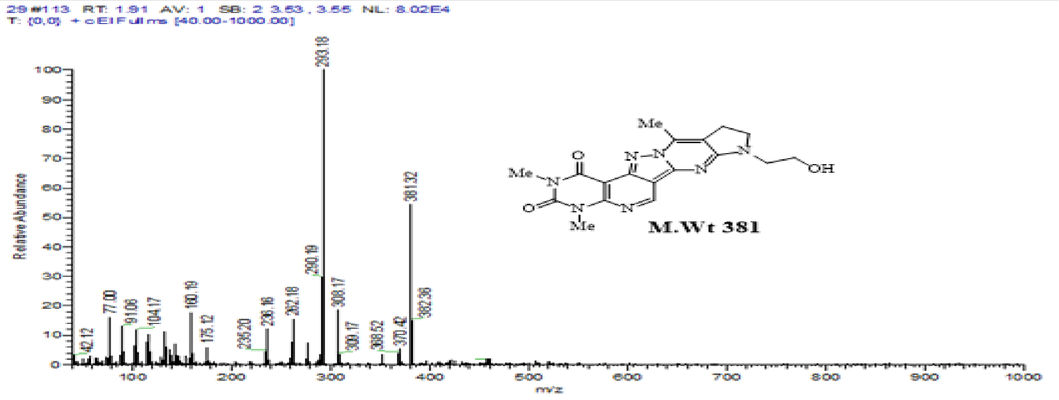

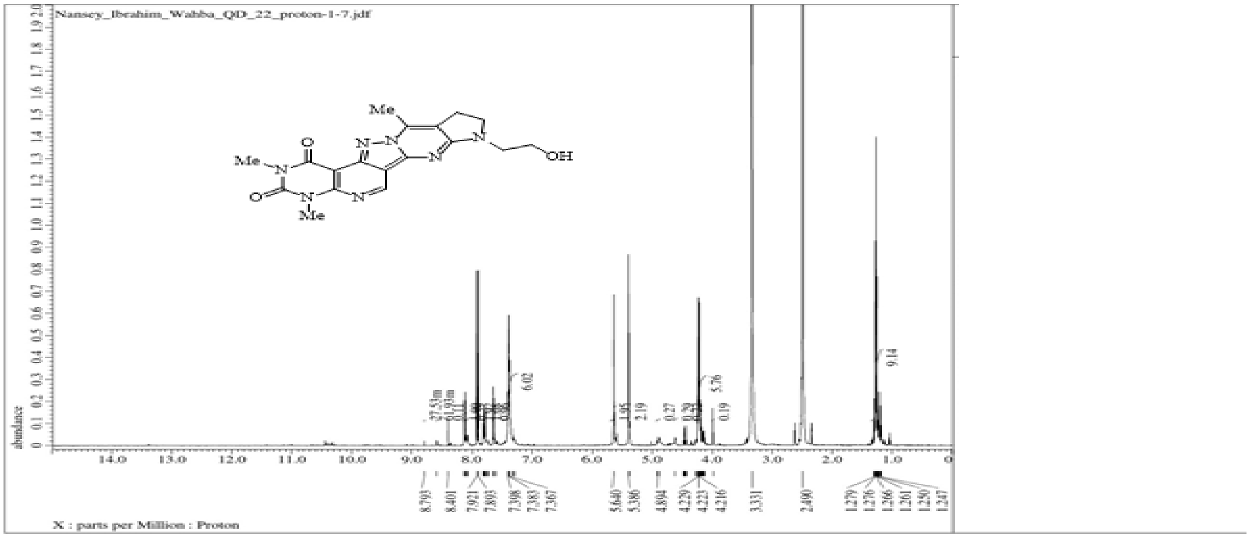

MS and ^{1}H NMR for Compound No. (7)

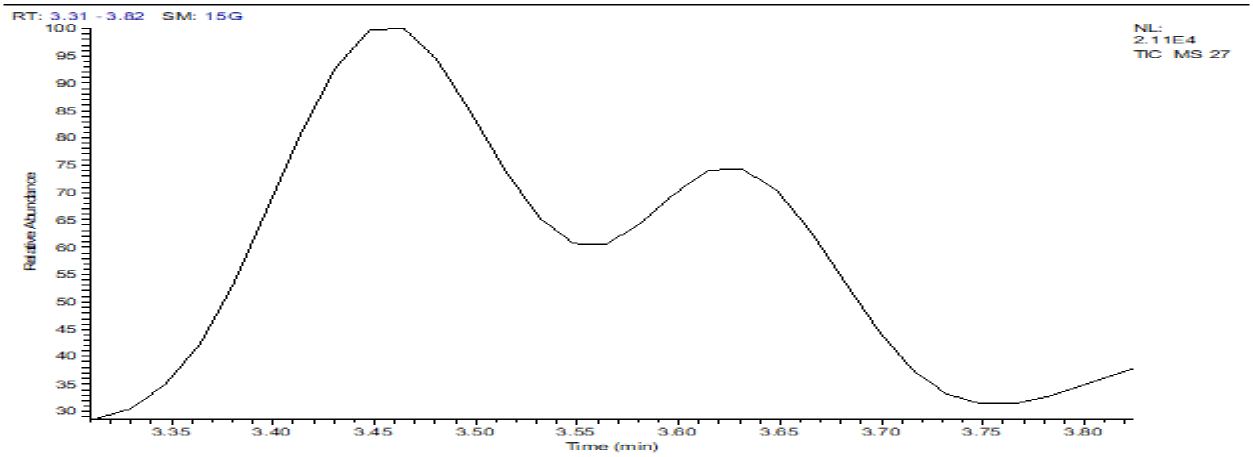

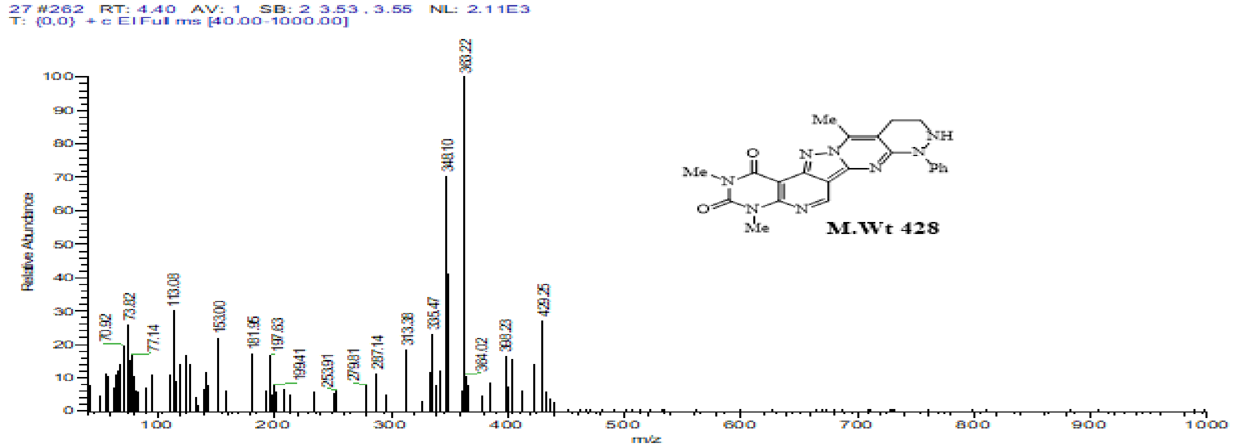

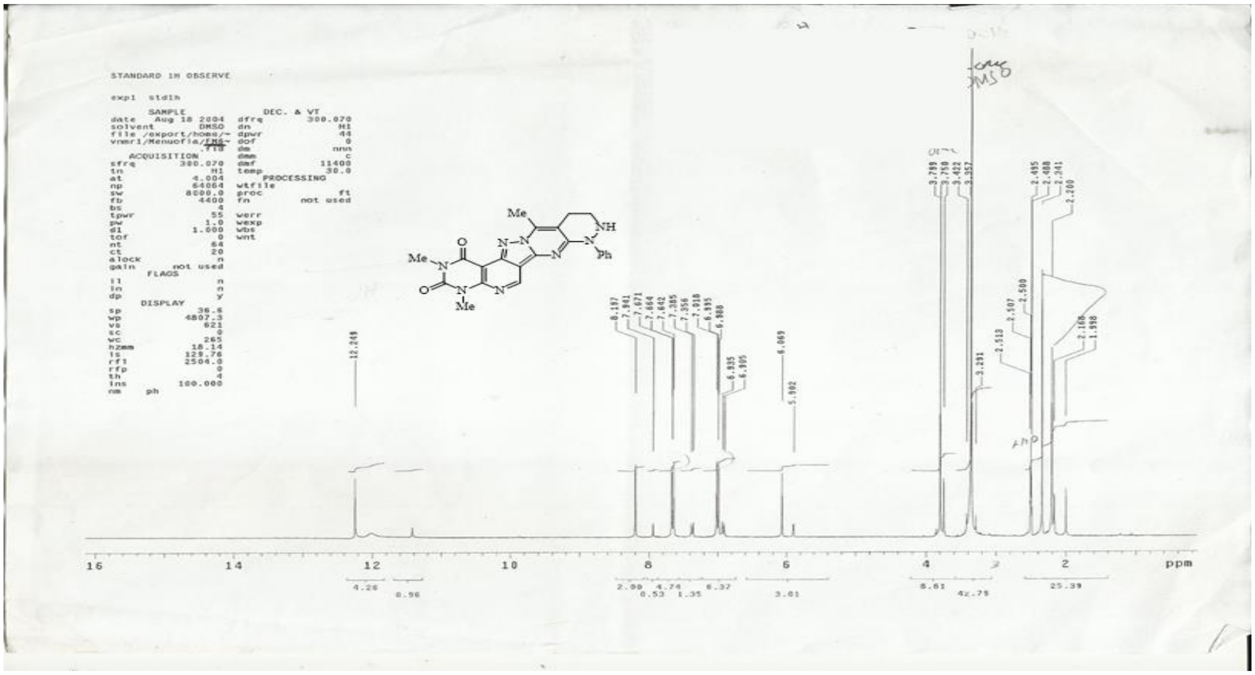

MS and ^{1}H NMR for Compound no (8)

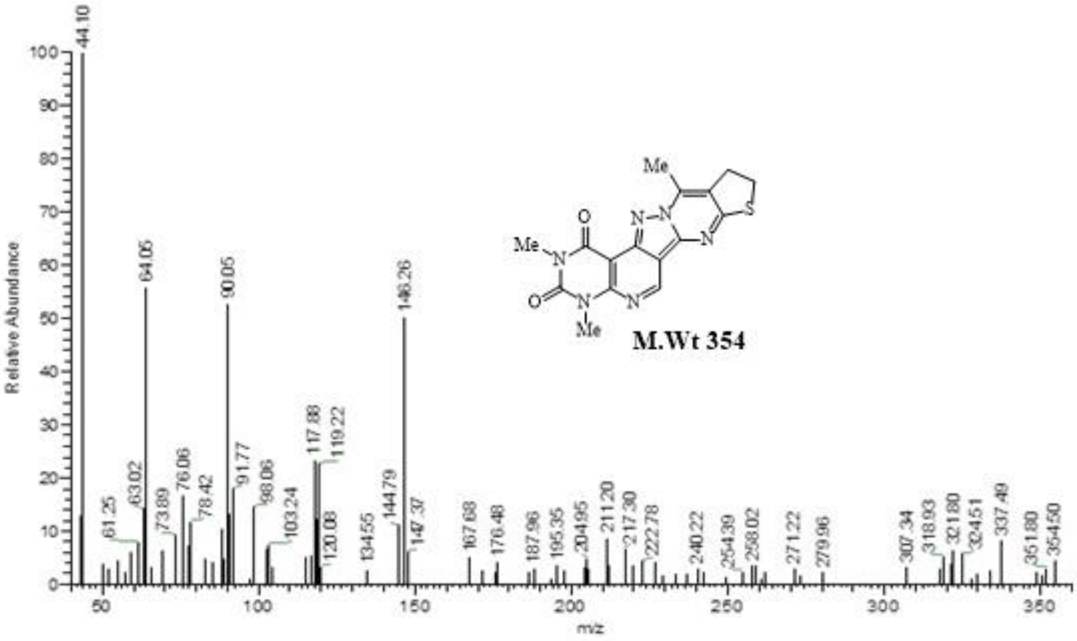

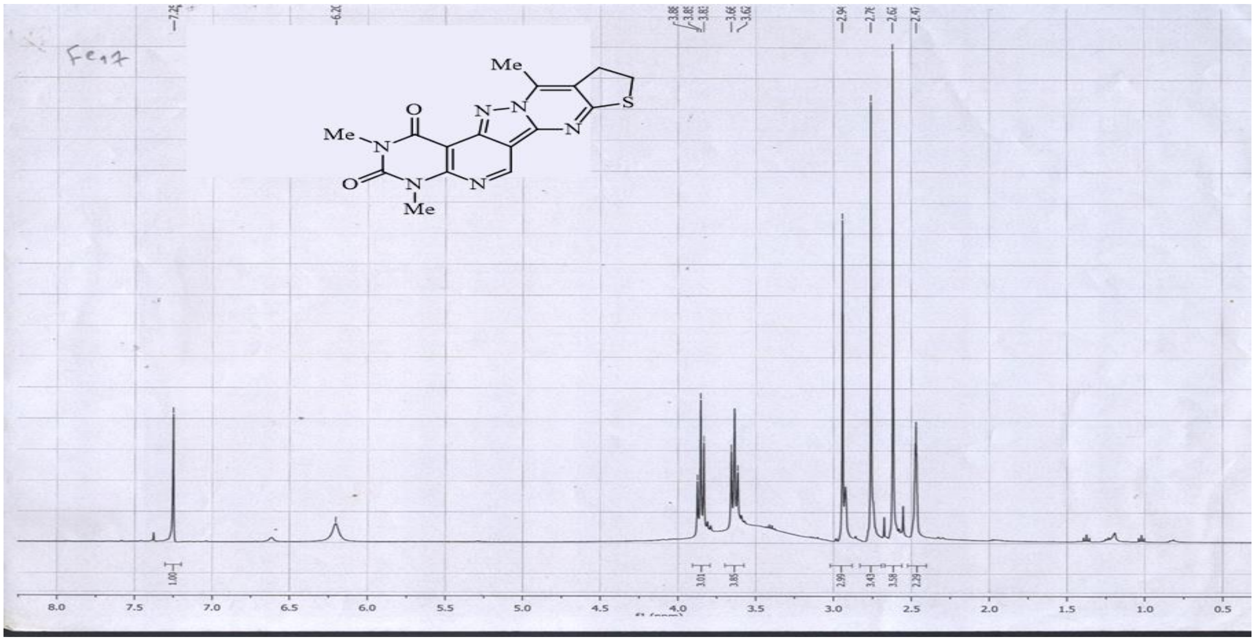

Supplement: Supplementary file 1 — Supporting Information [file OPEN-13-e202400070-s001.pdf]
